# The crystal structures, Hirshfeld surface analyses and energy frameworks of 8-{1-[3-(cyclo­pent-1-en-1-yl)benz­yl]piperidin-4-yl}-2-meth­oxy­quinoline and 8-{4-[3-(cyclo­pent-1-en-1-yl)benz­yl]piperazin-1-yl}-2-meth­oxy­quinoline

**DOI:** 10.1107/S2056989021002474

**Published:** 2021-03-09

**Authors:** Nisar Ullah, Helen Stoeckli-Evans

**Affiliations:** a Chemistry Department, King Fahd University of Petroleum and Minerals, Dhahran-31261, Saudi Arabia; b Institute of Physics, University of Neuchâtel, rue Emile-Argand 11, CH-2000 Neuchâtel, Switzerland

**Keywords:** crystal structure, meth­oxy­quinoline, piperidine, piperazine, cyclo­pentene, isoelectronic, isotypic, dopamine D_2_, serotonin 5-HT_1a_, hydrogen bonding, Hirshfeld surface analysis, energy frameworks

## Abstract

The title compounds, 8-{1-[3-(cyclo­pent-1-en-1-yl)benz­yl]piperidin-4-yl}-2-meth­oxy­quinoline, C_27_H_30_N_2_O (**I**), and 8-{4-[3-(cyclo­pent-1-en-1-yl)benz­yl]piperazin-1-yl}-2-meth­oxy­quinoline, C_26_H_29_N_3_O (**II**), differ only in the nature of the central six-membered ring: piperidine in **I** and piperazine in **II**. They are isoelectronic (CH *cf*. N) and isotypic; they both crystallize in the triclinic space group *P*  with very similar unit-cell parameters. Both mol­ecules have a curved shape and very similar conformations. In the biaryl group, the phenyl ring is inclined to the cyclo­pentene mean plane (r.m.s. deviations = 0.089 Å for **I** and 0.082 Å for **II**) by 15.83 (9) and 13.82 (6)° in **I** and **II**, respectively, and by 67.68 (6) and 69.47 (10)°, respectively, to the mean plane of the quinoline moiety (r.m.s. deviations = 0.034 Å for **I** and 0.038 Å for **II**). The piperazine ring in **I** and the piperidine ring in **II** have chair conformations. In the crystals of both compounds, mol­ecules are linked by C—H⋯π inter­actions, forming chains in **I** and ribbons in **II**, both propagating along the *b*-axis direction. The principal contributions to the overall Hirshfeld surfaces involve H⋯H contacts at 67.5 and 65.9% for **I** and **II**, respectively. The major contribution to the inter­molecular inter­actions in the crystals is from dispersion forces (*E*_dis_), reflecting the absence of classical hydrogen bonds.

## Abstract 2

The title compounds, 8-{1-[3-(cyclo­pent-1-en-1-yl)benz­yl]piperidin-4-yl}-2-meth­oxy­quinoline and 8-{4-[3-(cyclo­pent-1-en-1-yl)benz­yl]piperazin-1-yl}-2-meth­oxy­quinoline differ only in the nature of the central six-membered ring: piperidine in the first and piperazine in the second. They are isoelectronic (CH *cf*. N) and isotypic. The major contribution to the inter­molecular inter­actions in the crystals is from dispersion forces (*E*_dis_), reflecting the absence of classical hydrogen bonds.

## Chemical context   

Compounds combining dopamine D_2_ receptor blockade with serotonin 5-HT_1A_ receptor activation rather than antagonism for the treatment of Schizophrenia have been developed by a number of researchers (Newman-Tancredi *et al.*, 2007 ▸; Jones & McCreary, 2008 ▸). One such drug, Adoprazine^(c)^, was found to combine both dopamine D_2_ antagonist (blockade) and serotonin 5-HT_1A_ agonist (activation) properties (Feenstra *et al.*, 2001 ▸, 2006 ▸). A similar compound structurally, Bifeprunox^(c)^, is a partial agonist at dopamine D_2_ receptors *in vitro*, and shows serotonin 5-HT_1A_ agonist properties (Newman-Tancredi *et al.*, 2005 ▸; Cosi *et al.*, 2006 ▸). Unfortunately, development of Adoprazine^(c)^ was stopped at the Phase II clinical trials for insufficient therapeutical efficacy, and the FDA refused a licence for Bifeprunox^(c)^ for the same reason.

Ullah and collaborators have synthesized a series of compounds that are analogues of Adoprazine^(c)^ and Bifeprunox^(c)^ (Ullah, 2012 ▸, 2014*a* ▸,*b* ▸; Ullah & Al-Shaheri, 2012 ▸). They have examined rat-cloned dopamine D_2_ and human-cloned serotonin 5-HT_1A_ receptor properties of more than forty compounds (Ghani *et al.*, 2014 ▸; Ullah, 2014*a* ▸,*b* ▸), including the title compounds, 8-{1-[3-(cyclo­pent-1-en-1-yl)benz­yl]piperidin-4-yl}-2-meth­oxy­quinoline (**I**) and 8-{4-[3-(cyclo­pent-1-en-1-yl)benz­yl]piperazin-1-yl}-2-meth­oxy­quinoline (**II**). The D_2_ receptor binding assay of compounds **I** and **II** gave *K*_i_ = 524 nM for **I** and 12.2 nM for **II**. In the 5-HT_1A_ receptor binding assay, *K*_i_ = 2.13 nM for **I** and 0.97 nM for **II** (Ghani *et al.*, 2014 ▸). Replacing the piperidine ring in **I** with a piperazine ring in **II**, also present in Adoprazine^(c)^ and Bifeprunox^(c)^, has a significant effect and appears to be favourable for higher binding affinity.
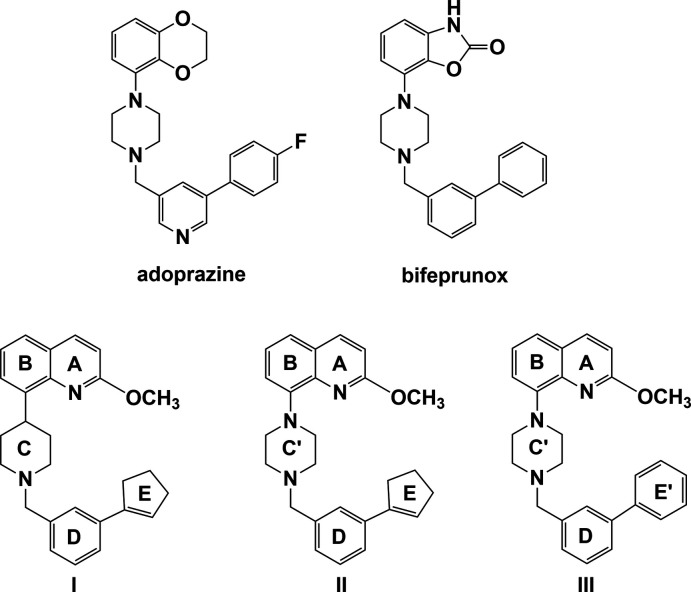


The crystal structure of **II** is compared to that of 8-[4-([1,1′-biphen­yl]-3-ylmeth­yl)piperazin-1-yl]-2-meth­oxy­quinoline (**III**), where the 3-(cyclo­pent-1-en-1-yl)benzyl unit in **II** has been replaced by a 1,1′-biphenyl unit in **III** (Ullah & Altaf, 2014 ▸).

## Structural commentary   

**Figure 1:**
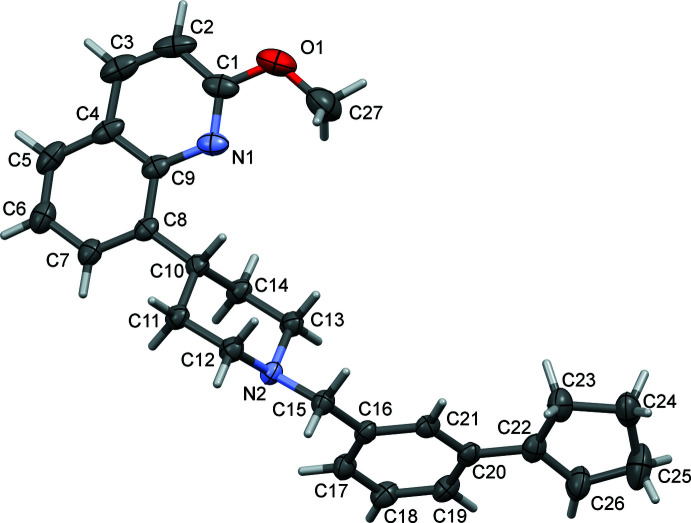
A view of the mol­ecular structure of **I**, with atom labelling. The displacement ellipsoids are drawn at the 50% probability level.

**Figure 3:**
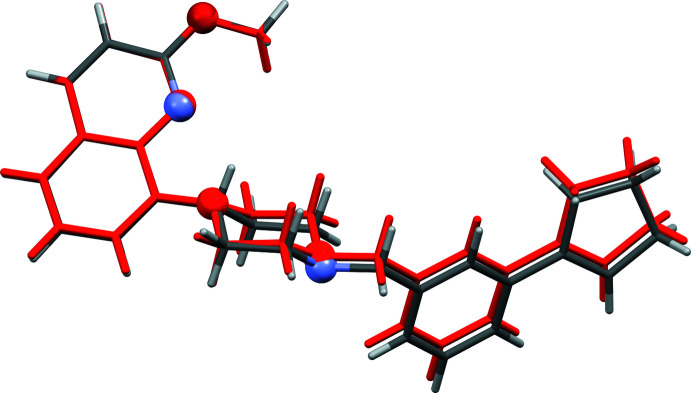
A view of the structural overlap of compounds **I** and **II** (red); r.m.s. deviation 0.002 Å (Mercury; Macrae *et al.*, 2020 ▸). The O and N atoms are shown as balls.

The mol­ecular structures of compounds **I** and **II** are shown in Figs. 1 ▸ and 2 ▸, respectively. They have very similar conformations, as illustrated by the view of their structural overlap, shown in Fig. 3 ▸. Both compounds crystallize in the triclinic space group *P* 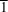 with very similar unit-cell parameters in spite of replacing the piperidine ring in **I** with a piperazine ring in **II**; they are isotypic and isoelectronic (CH *cf*. N). Both mol­ecules have a curved shape, and the piperidine ring (*C* = N2/C10–C14) in **I** and the piperazine ring (*C*′ = N2/N3/C10–C13) in **II** have chair conformations.

In the biaryl group, the phenyl ring (*D* = C16–C21 in **I** and C15–C20 in **II**) is inclined to the cyclo­pentene ring mean plane (*E* = C22–C26, r.m.s. deviation = 0.089 Å for **I** and *E* = C21–C25, r.m.s. deviation = 0.082 Å for **II**) by 15.83 (9) and 13.82 (16)°, respectively. The same ring *D* is inclined to the mean plane of the quinoline moiety (r.m.s. deviation = 0.034 Å for **I** and 0.038 Å for **II**) by 67.68 (6) and 69.47 (10)°, respectively. In the cyclo­pentene rings, the double bonds C22=C26 in **I** and C21=C25 in **II** are 1.381 (2) and 1.365 (4) Å, respectively, while bonds C22—C23 and C21—C22 are 1.450 (2) and 1.457 (4) Å, respectively. These values fall within the limits of those observed for the structures of 40 compounds in the Cambridge Structural Database (CSD, Version 5.42, last update February 2021; Groom *et al.*, 2016 ▸), *viz*. C=C varies from *ca* 1.268 to 1.417 Å, while the adjacent substituted C—C bond varies from *ca* 1.391 to 1.534 Å (see supporting information file S1).

**Figure 4:**
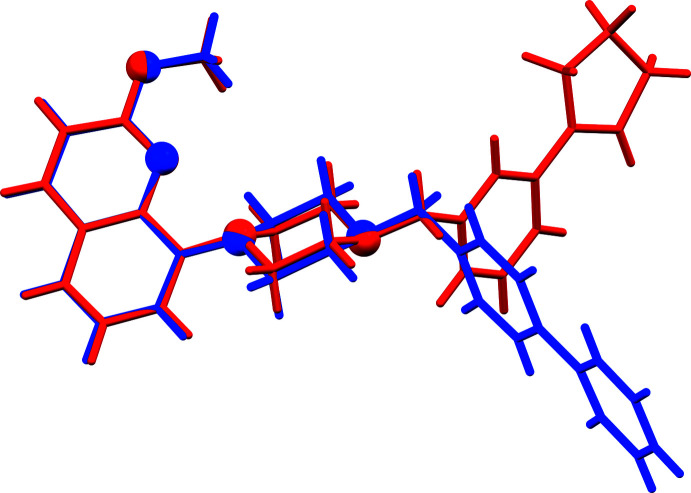
A view of the structural overlap of compounds **II** (red) and **III** (blue; Ullah & Altaf, 2014 ▸); r.m.s. deviation 0.071 Å (*Mercury*; Macrae *et al.*, 2020 ▸). The O and N atoms are shown as balls.

In compound **III**, the 3-(cyclo­pent-1-en-1-yl)benzyl unit in **II** has been replaced by a 1,1′-biphenyl group (supporting information file S2; Fig. S1). The conformation of the mol­ecules differs considerably, as illustrated in the view of their structural overlap (Fig. 4 ▸). The mol­ecule has an S-shape and torsion angles C12—N3—C14—C15 and N3—C14—C15—C16 are, respectively, −172.77 (16) and 61.9 (3)°, compared to −67.4 (3) and −43.2 (3) ° in **II**. As in **II**, the central piperazine ring (*C*′) has a chair conformation. The two rings of the biphenyl unit (rings *D* and *E*′) are relatively coplanar with a dihedral angle of 3.84 (12)°. Phenyl ring *D* is inclined to the mean plane of the quinoline ring system(r.m.s. deviation = 0.021 Å) by 68.94 (10)°, compared to 69.47 (10)° in **II**.

## Supra­molecular features   

**Figure 5:**
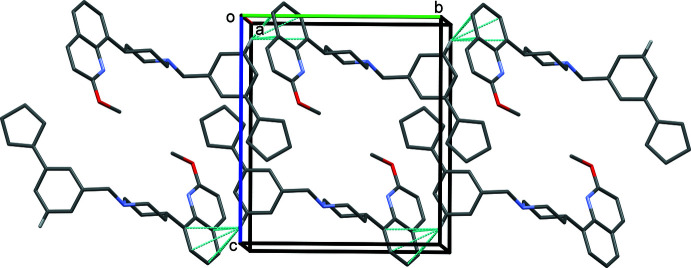
A view along the *a* axis of the crystal packing of **I**. The C—H⋯π inter­actions are shown as dashed lines (see Table 1 ▸). Only the H atoms involved in these inter­actions have been included.

**Figure 6:**
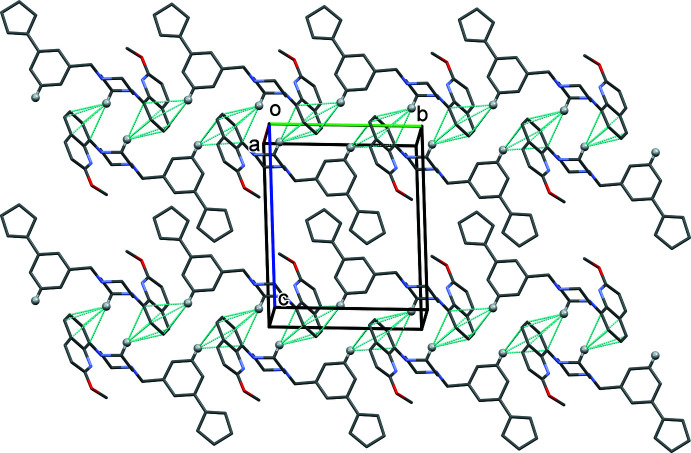
A view along the *a* axis of the crystal packing of **II**. The C—H⋯π inter­actions are shown as dashed lines (see Table 2 ▸). Only the H atoms involved in these inter­actions have been included.

In the crystals of **I** and **II**, mol­ecules are linked by C—H⋯π inter­actions (Tables 1 ▸ and 2 ▸, respectively). In **I**, a single C—H⋯π inter­action links the mol­ecules, forming chains propagating along the *b*-axis direction (Fig. 5 ▸). In **II**, two C—H⋯π inter­actions link the mol­ecules, forming ribbons propagating along the *b*-axis direction (Fig. 6 ▸). There are no other significant directional inter-atomic contacts present in either crystal structure.

In the crystal of **III**, mol­ecules are linked by C—H⋯O hydrogen bonds, forming chains along the [100] direction. The chains are linked by two C—H⋯π inter­actions, forming slabs lying parallel to the *ab* plane (supporting information file S2; Table S1 and Fig. S2). Here too, there are no other significant directional inter-atomic contacts present in the crystal structure.

## Hirshfeld surface analysis and two-dimensional fingerprint plots   

The Hirshfeld surface analysis (Spackman & Jayatilaka, 2009 ▸) and the associated two-dimensional fingerprint plots (McKinnon *et al.*, 2007 ▸) were performed with *CrystalExplorer17* (Turner *et al.*, 2017 ▸) following the protocol of Tiekink and collaborators (Tan *et al.*, 2019 ▸).

**Figure 7:**
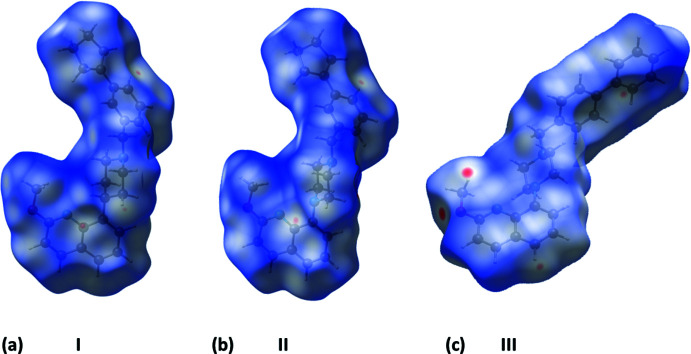
The Hirshfeld surfaces of compounds (*a*) **I**, (*b*) **II** and (c) **III**, mapped over *d*_norm_ in the colour ranges of −0.1177 to 1.5125, −0.2113 to 1.3756 and −0.1475 to 1.8614 au., respectively.

The Hirshfeld surfaces are colour-mapped with the normalized contact distance, *d*_norm_, varying from red (distances shorter than the sum of the van der Waals radii) through white to blue (distances longer than the sum of the van der Waals radii). The Hirshfeld surfaces (HS) of **I**, **II** and **III** mapped over *d*_norm_ are given in Fig. 7 ▸. The most significant short contacts in the crystal structures of all three compounds are given in Table 3 ▸. It is evident from the small red spots in Fig. 7 ▸ *a* and *b* that there are only weak contacts present in the crystals of compounds **I** and **II**. The slightly larger red spots in Fig. 7 ▸ *c* concern the C_ar_—H⋯O_meth­oxy_ hydrogen bonds in the crystal structure of **III** (supporting information Table S2).

**Figure 8:**
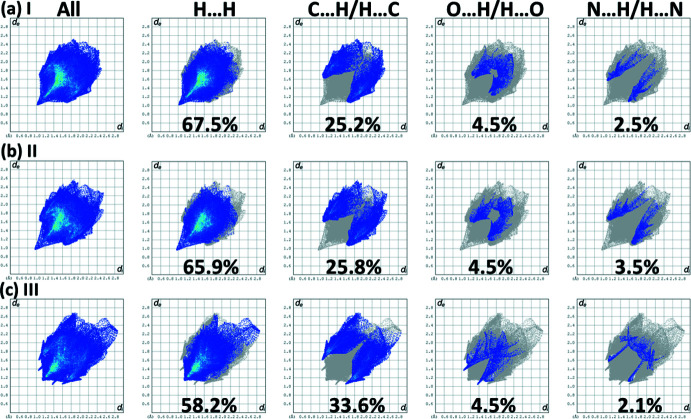
The full two-dimensional fingerprint plots for compounds (*a*) **I**, (*b*) **II** and (*c*) **III**, and those delineated into H⋯H, C⋯H/H⋯C, O⋯H/H⋯O and N⋯H/H⋯N contacts.

The percentage contributions of inter-atomic contacts to the HS for all three compounds are compared in Table 4 ▸. The two-dimensional fingerprint plots for compounds **I**, **II** and **III** are shown in Fig. 8 ▸. They reveal, as expected in the absence of classical hydrogen bonds, that the principal contributions to the overall HS surface involve H⋯H contacts at 67.5, 65.9 and 58.2%, respectively.

The second most important contribution to the HS is from the C⋯H/H⋯C contacts at 25.2, 25.8 and 33.6%, for **I**, **II** and **III**, respectively, which are related to the presence of C—H⋯π inter­actions (see Tables 1 ▸, 2 ▸ and S1). These are followed by O⋯H/H⋯O contacts at 4.5% for each compound. These two contributions are particularly significant for **III**, as indicated by the pair of sharp spikes for the delineated C⋯H/H⋯C and O⋯H/H⋯O contacts shown in Fig. 8 ▸ *c*.

The N⋯H/H⋯N contacts contribute, respectively, 2.5, 3.5 and 2.1%. The C⋯N contacts contribute even less; 1.3% in **III** but 0% in **I** and **II**. The C⋯C and C⋯O contacts contribute very little for all three structures.

**Figure 9:**
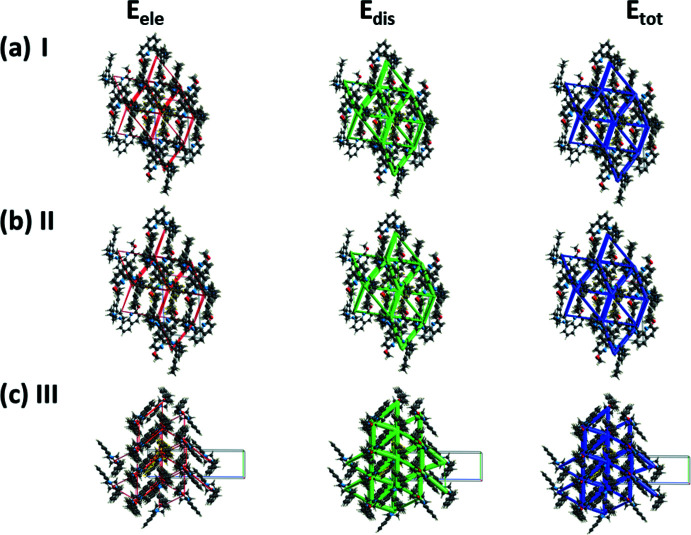
The energy frameworks calculated for (*a*) **I** and (*b*) **II**, both viewed along the *b* axis direction, and (*c*) **III**, viewed along the *a-*axis direction, showing the electrostatic potential forces (*E*_ele_), the dispersion forces (*E*_dis_) and the total energy diagrams (*E*_tot_).

The fact that compounds **I** and **II** are isoelectronic and isotypic is reflected in their almost identical Hirshfeld surfaces (Fig. 7 ▸ *a* and *b*), contributions of the inter-atomic contacts to the HS (Table 4 ▸), fingerprint plots (Fig. 8 ▸ *a* and *b*), and energy frameworks (Fig. 9 ▸ *a* and *b*).

## Energy frameworks   

A comparison of the energy frameworks calculated for **I**, **II** and **III**, showing the electrostatic potential forces (*E*_ele_), the dispersion forces (*E*_dis_) and the total energy diagrams (*E*_tot_), are shown in Fig. 9 ▸. The energies were obtained by using the wave function at the HF/3-2IG level of theory. The cylindrical radii are proportional to the relative strength of the corres­ponding energies (Turner *et al.*, 2017 ▸; Tan *et al.*, 2019 ▸). They have been adjusted to the same scale factor of 80 with a cut-off value of 5 kJ mol^−1^ within a radius of 6 Å of a central reference mol­ecule. It can be seen that for all three compounds, the major contribution to the inter­molecular inter­actions is from dispersion forces (*E*_dis_), reflecting the absence of classical hydrogen bonds in the crystals.

The colour-coded inter­action mappings within a radius of 6 Å of a central reference mol­ecule for all three compounds are given in the supporting information file S3. Full details of the various contributions to the total energy (*E*_tot_) are also included there.

## Database survey   

A search of the Cambridge Structural Database (CSD, Version 5.42, last update February 2021; Groom *et al.*, 2016 ▸) for 2-meth­oxy­quinolines gave 53 hits. In the majority of cases, the meth­oxy group (atoms C_ar_–O–C) lies close to the mean plane of the quinoline ring, with dihedral angles varying from 0 to *ca* 8.51°. In compounds **I**, **II** and **III** the same dihedral angles are 7.24 (16), 7.1 (2) and 1.98 (19)°, respectively. A search for 2-meth­oxy­quinolines with a piperidine or piperazine ring in the 8-position gave only one hit, *viz*. for compound **III** (CSD refcode: AKUXIQ; Ullah & Altaf, 2014 ▸).

## Synthesis and crystallization   

The synthesis of compounds **I**, **II** and **III** have been reported [**I** (Ullah & Al-Shaheri, 2012 ▸), compound **3** ***e*** in that paper; **II** and **III** (Ullah, 2012 ▸), compounds **3** ***e*** and **3** ***a***, respectively, in that paper]. Colourless crystals of **I** and **II** were obtained by slow evaporation of solutions in di­chloro­methane and methanol; ratios (8:3) and (8.5:1.5), respectively.

## Refinement   

Crystal data, data collection and structure refinement details are summarized in Table 5 ▸. For both compounds, the C-bound H atoms were included in calculated positions and refined as riding on the parent atom: C—H = 0.95–0.99 Å with *U*_iso_(H) = 1.5*U*_eq_(C-meth­yl) and *U*_iso_(H) = 1.2*U*_eq_(C) for other H atoms.

## Supplementary Material

Crystal structure: contains datablock(s) I, II, Global. DOI: 10.1107/S2056989021002474/zv2005sup1.cif

Structure factors: contains datablock(s) I. DOI: 10.1107/S2056989021002474/zv2005Isup2.hkl

Supporting information file. DOI: 10.1107/S2056989021002474/zv2005Isup4.cml

Structure factors: contains datablock(s) II. DOI: 10.1107/S2056989021002474/zv2005IIsup3.hkl

Supporting information file. DOI: 10.1107/S2056989021002474/zv2005IIsup5.cml

CSD search and analysis in Mercury of cyclopentene rings. DOI: 10.1107/S2056989021002474/zv2005sup6.pdf

Details concerning compound III. DOI: 10.1107/S2056989021002474/zv2005sup7.pdf

Colour-coded interaction mappings for compounds I, II and III. DOI: 10.1107/S2056989021002474/zv2005sup8.pdf

CCDC references: 997191, 997192

Additional supporting information:  crystallographic information; 3D view; checkCIF report

## Figures and Tables

**Figure 2:**
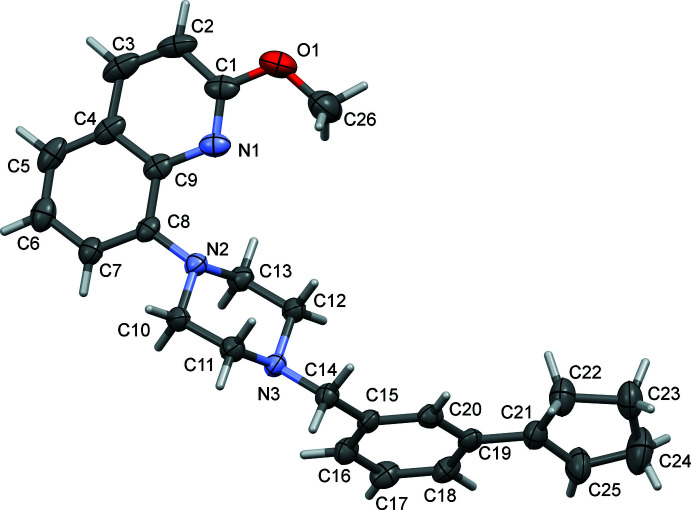
A view of the mol­ecular structure of **II**, with atom labelling. The displacement ellipsoids are drawn at the 50% probability level.

**Table 1:** Hydrogen-bond geometry (Å, °) for **I** [Chem scheme1] *CgB* is the centroid of ring C4–C9.

*D*—H⋯*A*	*D*—H	H⋯*A*	*D*⋯*A*	*D*—H⋯*A*
C18—H18⋯CgB^i^	0.95	2.97	3.661 (2)	131

Symmetry code: (i) x, y+1, z.

**Table 2:** Hydrogen-bond geometry (Å, °) for **II** [Chem scheme1] *CgB* is the centroid of ring C4-C9.

*D*—H⋯*A*	*D*—H	H⋯*A*	*D*⋯*A*	*D*—H⋯*A*
C13—H13*B*⋯CgB^i^	0.99	2.95	3.757 (3)	140
C17—H17⋯CgB^ii^	0.95	2.93	3.602 (3)	129

Symmetry codes: (i) -x, -y, -z; (ii) x, y+1, z.

**Table 3:** Table 3 ▸. Short contacts (Å) in the crystal structures of compounds **I**, **II** and **III** *^*a*^*

Atom1	Atom2	Length	Length − VdW	Symm. op. 1	Symm. op. 2
**I**					
H11*B*	H11*B*	2.187	−0.213	*x*, *y*, *z*	1 − *x*, 1 − *y*, −*z*
C9	H19	2.827	−0.073	*x*, *y*, *z*	*x*, −1 + *y*, *z*
H2	C19	2.834	−0.066	*x*, *y*, *z*	−1 + *x*, −1 + *y*, *z*
C3	H11*A*	2.843	−0.057	*x*, *y*, *z*	−1 + *x*, *y*, *z*
C2	H11*A*	2.865	−0.035	*x*, *y*, *z*	−1 + *x*, *y*, *z*
H2	C20	2.904	0.004	*x*, *y*, *z*	−1 + *x*, −1 + *y*, *z*
					
**II**					
H10*A*	H10*A*	2.076	−0.324	*x*, *y*, *z*	1 − *x*, −*y*, −*z*
H2	C18	2.824	−0.076	*x*, *y*, *z*	−1 + *x*, −1 + *y*, *z*
C8	H18	2.824	−0.076	*x*, *y*, *z*	*x*, −1 + *y*, *z*
C9	H18	2.867	−0.033	*x*, *y*, *z*	*x*, −1 + *y*, *z*
C10	H10*A*	2.866	−0.034	*x*, *y*, *z*	1 − *x*, −*y*, −*z*
C3	H10*B*	2.878	−0.022	*x*, *y*, *z*	−1 + *x*, *y*, *z*
C1	C11	3.403	0.003	*x*, *y*, *z*	−1 + *x*, *y*, *z*
					
**III** *^*a*^*					
O1	H24	2.514	−0.206	*x*, *y*, *z*	−1 + *x*, *y*, *z*
C4	H27*B*	2.741	−0.159	*x*, *y*, *z*	*x*, −1 + *y*, *z*
C21	H5	2.826	−0.074	*x*, *y*, *z*	2 − *x*, 1 − *y*, 2 − *z*
H12*A*	C27	2.845	−0.055	*x*, *y*, *z*	{3\over 2} − *x*, −{1\over 2} + *y*, {3\over 2} − *z*
O1	H14*B*	2.722	0.002	*x*, *y*, *z*	{3\over 2} − *x*, −{1\over 2} + *y*, {3\over 2} − *z*

Note: (*a*) Ullah & Altaf (2014 ▸).

**Table 4:** Principal percentage contributions of inter-atomic contacts to the Hirshfeld surfaces of **I**, **II** and **III** *^*a*^*

Contact	**I**	**II**	**III** *^*a*^*
H⋯H	67.5	65.9	58.2
C⋯H/H⋯C	25.2	25.8	33.6
O⋯H/H⋯O	4.5	4.5	4.5
N⋯H/H⋯N	2.5	3.5	2.1
C⋯C	0.2	0.2	0.2
C⋯N	0	0	1.3
C⋯O	0.1	0.1	0.1

Note: (*a*) Ullah & Altaf (2014 ▸).

**Table 5:** Experimental details

	**I**	**II**
Crystal data		
Chemical formula	C_27_H_30_N_2_O	C_26_H_29_N_3_O
*M*_r_	398.53	399.52
Crystal system, space group	Triclinic, *P*\overline{1}	Triclinic, *P*\overline{1}
Temperature (K)	173	173
*a*, *b*, *c* (Å)	7.7099 (7), 11.2838 (10), 12.9539 (13)	7.9142 (8), 10.9051 (13), 12.8896 (14)
α, β, γ (°)	89.413 (8), 79.094 (7), 82.270 (7)	87.271 (9), 79.290 (8), 82.206 (9)
*V* (Å^3^)	1096.41 (18)	1082.7 (2)
*Z*	2	2
Radiation type	Mo *K*α	Mo *K*α
μ (mm^−1^)	0.07	0.08
Crystal size (mm)	0.45 × 0.37 × 0.25	0.45 × 0.40 × 0.19

Data collection		
Diffractometer	Stoe IPDS 2	Stoe IPDS 2
Absorption correction	Multi-scan (*MULABS*; Spek, 2020 ▸)	Multi-scan (*MULABS*; Spek, 2020 ▸)
*T*_min_, *T*_max_	0.897, 1.000	0.793, 1.000
No. of measured, independent and observed [*I* > 2σ(*I*)] reflections	14097, 4138, 2585	11304, 4088, 2187
*R*_int_	0.038	0.080
(sin θ/λ)_max_ (Å^−1^)	0.609	0.610

Refinement		
*R*[*F* ^2^ > 2σ(*F* ^2^)], *wR*(*F* ^2^), *S*	0.035, 0.084, 0.83	0.056, 0.121, 0.90
No. of reflections	4138	4088
No. of parameters	273	273
No. of restraints	0	1
H-atom treatment	H-atom parameters constrained	H-atom parameters constrained
Δρ_max_, Δρ_min_ (e Å^−3^)	0.31, −0.19	0.28, −0.17

Computer programs: *X-AREA* and *X-RED32* (Stoe & Cie, 2009 ▸), *SHELXS97* (Sheldrick, 2008 ▸), *SHELXL2018/3* (Sheldrick, 2015 ▸), *PLATON* (Spek, 2020 ▸, *Mercury* (Macrae *et al.*, 2020 ▸) and *publCIF* (Westrip, 2010 ▸).

## supplementary crystallographic information

### 8-{1-[3-(Cyclopent-1-en-1-yl)benzyl]piperidin-4-yl}-2-methoxyquinoline (I) Crystal data

**Table:**

C_27_H_30_N_2_O	*Z* = 2
*M**_r_* = 398.53	*F*(000) = 428
Triclinic, *P*1	*D*_x_ = 1.207 Mg m^−^^3^
*a* = 7.7099 (7) Å	Mo *K*α radiation, λ = 0.71073 Å
*b* = 11.2838 (10) Å	Cell parameters from 8113 reflections
*c* = 12.9539 (13) Å	θ = 1.6–26.1°
α = 89.413 (8)°	µ = 0.07 mm^−^^1^
β = 79.094 (7)°	*T* = 173 K
γ = 82.270 (7)°	Block, colourless
*V* = 1096.41 (18) Å^3^	0.45 × 0.37 × 0.25 mm

### 8-{1-[3-(Cyclopent-1-en-1-yl)benzyl]piperidin-4-yl}-2-methoxyquinoline (I) Data collection

**Table:**

STOE IPDS 2 diffractometer	4138 independent reflections
Radiation source: fine-focus sealed tube	2585 reflections with *I* > 2σ(*I*)
Plane graphite monochromator	*R*_int_ = 0.038
φ + ω scans	θ_max_ = 25.6°, θ_min_ = 1.8°
Absorption correction: multi-scan (MULABS; Spek, 2020)	*h* = −9→9
*T*_min_ = 0.897, *T*_max_ = 1.000	*k* = −13→13
14097 measured reflections	*l* = −15→15

### 8-{1-[3-(Cyclopent-1-en-1-yl)benzyl]piperidin-4-yl}-2-methoxyquinoline (I) Refinement

**Table:**

Refinement on *F*^2^	Secondary atom site location: difference Fourier map
Least-squares matrix: full	Hydrogen site location: inferred from neighbouring sites
*R*[*F*^2^ > 2σ(*F*^2^)] = 0.035	H-atom parameters constrained
*wR*(*F*^2^) = 0.084	*w* = 1/[σ^2^(*F*_o_^2^) + (0.0484*P*)^2^] where *P* = (*F*_o_^2^ + 2*F*_c_^2^)/3
*S* = 0.83	(Δ/σ)_max_ < 0.001
4138 reflections	Δρ_max_ = 0.31 e Å^−^^3^
273 parameters	Δρ_min_ = −0.18 e Å^−^^3^
0 restraints	Extinction correction: (*SHELXL2018/3*; Sheldrick, 2015), Fc^*^=kFc[1+0.001xFc^2^λ^3^/sin(2θ)]^-1/4^
Primary atom site location: structure-invariant direct methods	Extinction coefficient: 0.0094 (16)

### 8-{1-[3-(Cyclopent-1-en-1-yl)benzyl]piperidin-4-yl}-2-methoxyquinoline (I) Special details

**Table:**

Geometry. All esds (except the esd in the dihedral angle between two l.s. planes) are estimated using the full covariance matrix. The cell esds are taken into account individually in the estimation of esds in distances, angles and torsion angles; correlations between esds in cell parameters are only used when they are defined by crystal symmetry. An approximate (isotropic) treatment of cell esds is used for estimating esds involving l.s. planes.

### 8-{1-[3-(Cyclopent-1-en-1-yl)benzyl]piperidin-4-yl}-2-methoxyquinoline (I) Fractional atomic coordinates and isotropic or equivalent isotropic displacement parameters (Å^2^)

**Table:**

	*x*	*y*	*z*	*U*_iso_*/*U*_eq_	
O1	−0.31520 (16)	0.30564 (11)	0.39378 (9)	0.0592 (3)	
N1	−0.09673 (16)	0.31466 (10)	0.24743 (9)	0.0366 (3)	
N2	0.39949 (15)	0.61267 (9)	0.18470 (8)	0.0279 (3)	
C1	−0.2350 (2)	0.26704 (14)	0.29519 (13)	0.0446 (4)	
C2	−0.3120 (2)	0.17738 (15)	0.25111 (16)	0.0533 (5)	
H2	−0.409740	0.143721	0.290814	0.064*	
C3	−0.2430 (2)	0.14117 (14)	0.15136 (16)	0.0514 (5)	
H3	−0.293407	0.081958	0.119624	0.062*	
C4	−0.0953 (2)	0.19087 (12)	0.09322 (13)	0.0398 (4)	
C5	−0.0161 (2)	0.15780 (13)	−0.01128 (13)	0.0445 (4)	
H5	−0.065366	0.102091	−0.047870	0.053*	
C6	0.1305 (2)	0.20515 (13)	−0.06017 (13)	0.0429 (4)	
H6	0.182521	0.182977	−0.130942	0.051*	
C7	0.2054 (2)	0.28677 (12)	−0.00641 (11)	0.0361 (3)	
H7	0.309298	0.317564	−0.041440	0.043*	
C8	0.13307 (19)	0.32336 (11)	0.09513 (11)	0.0303 (3)	
C9	−0.02281 (19)	0.27612 (11)	0.14589 (11)	0.0338 (3)	
C10	0.21444 (18)	0.40881 (11)	0.15518 (10)	0.0291 (3)	
H10	0.191957	0.384932	0.230597	0.035*	
C11	0.41514 (18)	0.40490 (11)	0.12068 (11)	0.0322 (3)	
H11A	0.474251	0.322324	0.126794	0.039*	
H11B	0.442908	0.428067	0.046058	0.039*	
C12	0.48648 (19)	0.48939 (11)	0.18812 (11)	0.0323 (3)	
H12A	0.467098	0.462037	0.261762	0.039*	
H12B	0.616450	0.486966	0.163034	0.039*	
C13	0.20593 (18)	0.61870 (12)	0.21814 (11)	0.0323 (3)	
H13A	0.149684	0.702328	0.213674	0.039*	
H13B	0.178461	0.594316	0.292439	0.039*	
C14	0.12757 (19)	0.53857 (11)	0.15092 (11)	0.0332 (3)	
H14A	0.147602	0.565916	0.077284	0.040*	
H14B	−0.002595	0.543682	0.176720	0.040*	
C15	0.47093 (19)	0.68831 (11)	0.25299 (10)	0.0310 (3)	
H15A	0.602393	0.678391	0.231762	0.037*	
H15B	0.442130	0.660363	0.326154	0.037*	
C16	0.40022 (18)	0.81994 (11)	0.25048 (10)	0.0289 (3)	
C17	0.38518 (19)	0.87725 (12)	0.15602 (11)	0.0339 (3)	
H17	0.416942	0.833137	0.091594	0.041*	
C18	0.3239 (2)	0.99862 (12)	0.15611 (11)	0.0371 (4)	
H18	0.314063	1.037213	0.091545	0.045*	
C19	0.27704 (19)	1.06377 (12)	0.24901 (11)	0.0352 (3)	
H19	0.234929	1.146760	0.247712	0.042*	
C20	0.29069 (18)	1.00935 (11)	0.34499 (11)	0.0303 (3)	
C21	0.35196 (18)	0.88669 (11)	0.34328 (10)	0.0296 (3)	
H21	0.360782	0.847843	0.407859	0.036*	
C22	0.24386 (19)	1.07783 (13)	0.44478 (11)	0.0353 (3)	
C23	0.2242 (2)	1.02292 (14)	0.54733 (12)	0.0479 (4)	
H23A	0.339477	0.978796	0.557619	0.057*	
H23B	0.135586	0.966088	0.553796	0.057*	
C24	0.1619 (2)	1.12327 (15)	0.62848 (13)	0.0535 (5)	
H24A	0.234142	1.114906	0.684369	0.064*	
H24B	0.035106	1.122668	0.661147	0.064*	
C25	0.1867 (3)	1.23818 (16)	0.56826 (15)	0.0723 (6)	
H25A	0.078370	1.297650	0.585603	0.087*	
H25B	0.289265	1.273337	0.584998	0.087*	
C26	0.2204 (2)	1.20096 (14)	0.45501 (13)	0.0526 (5)	
H26	0.225071	1.254655	0.397896	0.063*	
C27	−0.2468 (3)	0.40355 (19)	0.43470 (14)	0.0682 (6)	
H27C	−0.321335	0.429785	0.502637	0.102*	
H27B	−0.248148	0.469981	0.385397	0.102*	
H27A	−0.124284	0.377844	0.444035	0.102*	

### 8-{1-[3-(Cyclopent-1-en-1-yl)benzyl]piperidin-4-yl}-2-methoxyquinoline (I) Atomic displacement parameters (Å^2^)

**Table:**

	*U*^11^	*U*^22^	*U*^33^	*U*^12^	*U*^13^	*U*^23^
O1	0.0465 (7)	0.0720 (8)	0.0567 (8)	−0.0146 (6)	−0.0006 (6)	0.0222 (6)
N1	0.0313 (7)	0.0348 (6)	0.0441 (8)	−0.0057 (5)	−0.0078 (6)	0.0124 (6)
N2	0.0288 (6)	0.0232 (5)	0.0319 (6)	−0.0030 (5)	−0.0068 (5)	−0.0030 (5)
C1	0.0350 (9)	0.0479 (9)	0.0511 (10)	−0.0067 (7)	−0.0087 (8)	0.0216 (8)
C2	0.0372 (10)	0.0484 (10)	0.0812 (14)	−0.0197 (8)	−0.0207 (9)	0.0316 (9)
C3	0.0460 (10)	0.0363 (9)	0.0805 (13)	−0.0133 (8)	−0.0292 (10)	0.0149 (8)
C4	0.0372 (9)	0.0248 (7)	0.0633 (11)	−0.0061 (6)	−0.0240 (8)	0.0107 (7)
C5	0.0550 (11)	0.0267 (7)	0.0599 (11)	−0.0050 (7)	−0.0318 (9)	−0.0018 (7)
C6	0.0550 (11)	0.0321 (8)	0.0446 (9)	−0.0025 (7)	−0.0190 (8)	−0.0044 (7)
C7	0.0442 (9)	0.0273 (7)	0.0384 (8)	−0.0051 (6)	−0.0115 (7)	−0.0012 (6)
C8	0.0343 (8)	0.0212 (6)	0.0366 (8)	−0.0025 (6)	−0.0112 (6)	0.0035 (6)
C9	0.0359 (9)	0.0242 (7)	0.0433 (9)	−0.0029 (6)	−0.0138 (7)	0.0081 (6)
C10	0.0339 (8)	0.0239 (7)	0.0295 (7)	−0.0046 (6)	−0.0054 (6)	0.0016 (5)
C11	0.0334 (8)	0.0235 (7)	0.0387 (8)	−0.0013 (6)	−0.0064 (6)	−0.0022 (6)
C12	0.0305 (8)	0.0268 (7)	0.0390 (8)	−0.0003 (6)	−0.0074 (6)	−0.0005 (6)
C13	0.0297 (8)	0.0267 (7)	0.0388 (8)	−0.0008 (6)	−0.0038 (6)	−0.0035 (6)
C14	0.0307 (8)	0.0258 (7)	0.0433 (8)	−0.0026 (6)	−0.0081 (7)	−0.0019 (6)
C15	0.0365 (8)	0.0280 (7)	0.0298 (7)	−0.0045 (6)	−0.0092 (6)	−0.0019 (6)
C16	0.0291 (8)	0.0269 (7)	0.0327 (7)	−0.0069 (6)	−0.0083 (6)	−0.0023 (6)
C17	0.0397 (9)	0.0324 (7)	0.0311 (8)	−0.0066 (6)	−0.0094 (6)	−0.0036 (6)
C18	0.0451 (9)	0.0324 (8)	0.0381 (8)	−0.0083 (7)	−0.0170 (7)	0.0053 (6)
C19	0.0373 (9)	0.0262 (7)	0.0454 (9)	−0.0049 (6)	−0.0159 (7)	−0.0001 (6)
C20	0.0250 (7)	0.0295 (7)	0.0379 (8)	−0.0059 (6)	−0.0080 (6)	−0.0051 (6)
C21	0.0295 (8)	0.0292 (7)	0.0319 (8)	−0.0066 (6)	−0.0083 (6)	0.0010 (6)
C22	0.0297 (8)	0.0356 (8)	0.0416 (8)	−0.0007 (6)	−0.0113 (7)	−0.0080 (6)
C23	0.0545 (11)	0.0502 (10)	0.0390 (9)	−0.0037 (8)	−0.0108 (8)	−0.0090 (7)
C24	0.0506 (11)	0.0626 (11)	0.0459 (10)	−0.0050 (9)	−0.0063 (8)	−0.0198 (8)
C25	0.0945 (16)	0.0519 (11)	0.0676 (13)	0.0138 (11)	−0.0228 (12)	−0.0270 (10)
C26	0.0671 (12)	0.0368 (9)	0.0506 (10)	0.0005 (8)	−0.0076 (9)	−0.0114 (7)
C27	0.0631 (13)	0.0942 (15)	0.0441 (10)	−0.0159 (12)	0.0017 (9)	0.0036 (10)

### 8-{1-[3-(Cyclopent-1-en-1-yl)benzyl]piperidin-4-yl}-2-methoxyquinoline (I) Geometric parameters (Å, º)

**Table:**

O1—C1	1.358 (2)	C13—H13B	0.9900
O1—C27	1.433 (2)	C14—H14A	0.9900
N1—C1	1.3060 (19)	C14—H14B	0.9900
N1—C9	1.3804 (19)	C15—C16	1.5140 (18)
N2—C15	1.4631 (16)	C15—H15A	0.9900
N2—C12	1.4649 (16)	C15—H15B	0.9900
N2—C13	1.4656 (17)	C16—C21	1.3873 (18)
C1—C2	1.416 (2)	C16—C17	1.3951 (18)
C2—C3	1.346 (2)	C17—C18	1.3873 (19)
C2—H2	0.9500	C17—H17	0.9500
C3—C4	1.420 (2)	C18—C19	1.379 (2)
C3—H3	0.9500	C18—H18	0.9500
C4—C5	1.407 (2)	C19—C20	1.3966 (19)
C4—C9	1.416 (2)	C19—H19	0.9500
C5—C6	1.360 (2)	C20—C21	1.4001 (18)
C5—H5	0.9500	C20—C22	1.4721 (19)
C6—C7	1.405 (2)	C21—H21	0.9500
C6—H6	0.9500	C22—C26	1.381 (2)
C7—C8	1.3723 (19)	C22—C23	1.450 (2)
C7—H7	0.9500	C23—C24	1.518 (2)
C8—C9	1.424 (2)	C23—H23A	0.9900
C8—C10	1.5137 (19)	C23—H23B	0.9900
C10—C11	1.5220 (19)	C24—C25	1.522 (3)
C10—C14	1.5310 (18)	C24—H24A	0.9900
C10—H10	1.0000	C24—H24B	0.9900
C11—C12	1.5214 (19)	C25—C26	1.495 (2)
C11—H11A	0.9900	C25—H25A	0.9900
C11—H11B	0.9900	C25—H25B	0.9900
C12—H12A	0.9900	C26—H26	0.9500
C12—H12B	0.9900	C27—H27C	0.9800
C13—C14	1.5175 (19)	C27—H27B	0.9800
C13—H13A	0.9900	C27—H27A	0.9800
			
C1—O1—C27	116.01 (13)	C10—C14—H14A	109.6
C1—N1—C9	117.41 (13)	C13—C14—H14B	109.6
C15—N2—C12	109.07 (10)	C10—C14—H14B	109.6
C15—N2—C13	110.40 (10)	H14A—C14—H14B	108.1
C12—N2—C13	110.58 (10)	N2—C15—C16	114.10 (11)
N1—C1—O1	119.14 (15)	N2—C15—H15A	108.7
N1—C1—C2	124.76 (16)	C16—C15—H15A	108.7
O1—C1—C2	116.10 (15)	N2—C15—H15B	108.7
C3—C2—C1	118.13 (16)	C16—C15—H15B	108.7
C3—C2—H2	120.9	H15A—C15—H15B	107.6
C1—C2—H2	120.9	C21—C16—C17	118.61 (12)
C2—C3—C4	120.47 (16)	C21—C16—C15	119.97 (12)
C2—C3—H3	119.8	C17—C16—C15	121.41 (12)
C4—C3—H3	119.8	C18—C17—C16	120.03 (13)
C5—C4—C9	119.15 (14)	C18—C17—H17	120.0
C5—C4—C3	123.76 (15)	C16—C17—H17	120.0
C9—C4—C3	117.07 (15)	C19—C18—C17	120.64 (13)
C6—C5—C4	120.36 (14)	C19—C18—H18	119.7
C6—C5—H5	119.8	C17—C18—H18	119.7
C4—C5—H5	119.8	C18—C19—C20	120.86 (13)
C5—C6—C7	120.26 (15)	C18—C19—H19	119.6
C5—C6—H6	119.9	C20—C19—H19	119.6
C7—C6—H6	119.9	C19—C20—C21	117.63 (12)
C8—C7—C6	122.03 (15)	C19—C20—C22	121.62 (12)
C8—C7—H7	119.0	C21—C20—C22	120.75 (12)
C6—C7—H7	119.0	C16—C21—C20	122.23 (12)
C7—C8—C9	117.94 (13)	C16—C21—H21	118.9
C7—C8—C10	122.77 (13)	C20—C21—H21	118.9
C9—C8—C10	119.28 (12)	C26—C22—C23	110.50 (13)
N1—C9—C4	122.07 (14)	C26—C22—C20	125.83 (14)
N1—C9—C8	117.72 (13)	C23—C22—C20	123.61 (13)
C4—C9—C8	120.20 (14)	C22—C23—C24	106.97 (14)
C8—C10—C11	114.28 (11)	C22—C23—H23A	110.3
C8—C10—C14	112.62 (11)	C24—C23—H23A	110.3
C11—C10—C14	108.35 (11)	C22—C23—H23B	110.3
C8—C10—H10	107.1	C24—C23—H23B	110.3
C11—C10—H10	107.1	H23A—C23—H23B	108.6
C14—C10—H10	107.1	C23—C24—C25	105.45 (14)
C12—C11—C10	110.69 (11)	C23—C24—H24A	110.7
C12—C11—H11A	109.5	C25—C24—H24A	110.7
C10—C11—H11A	109.5	C23—C24—H24B	110.7
C12—C11—H11B	109.5	C25—C24—H24B	110.7
C10—C11—H11B	109.5	H24A—C24—H24B	108.8
H11A—C11—H11B	108.1	C26—C25—C24	104.67 (14)
N2—C12—C11	111.93 (11)	C26—C25—H25A	110.8
N2—C12—H12A	109.2	C24—C25—H25A	110.8
C11—C12—H12A	109.2	C26—C25—H25B	110.8
N2—C12—H12B	109.2	C24—C25—H25B	110.8
C11—C12—H12B	109.2	H25A—C25—H25B	108.9
H12A—C12—H12B	107.9	C22—C26—C25	110.74 (15)
N2—C13—C14	111.93 (11)	C22—C26—H26	124.6
N2—C13—H13A	109.2	C25—C26—H26	124.6
C14—C13—H13A	109.2	O1—C27—H27C	109.5
N2—C13—H13B	109.2	O1—C27—H27B	109.5
C14—C13—H13B	109.2	H27C—C27—H27B	109.5
H13A—C13—H13B	107.9	O1—C27—H27A	109.5
C13—C14—C10	110.27 (11)	H27C—C27—H27A	109.5
C13—C14—H14A	109.6	H27B—C27—H27A	109.5
			
C9—N1—C1—O1	−177.72 (13)	C13—N2—C12—C11	57.05 (14)
C9—N1—C1—C2	1.3 (2)	C10—C11—C12—N2	−57.40 (15)
C27—O1—C1—N1	4.3 (2)	C15—N2—C13—C14	−178.41 (11)
C27—O1—C1—C2	−174.73 (14)	C12—N2—C13—C14	−57.62 (14)
N1—C1—C2—C3	−2.6 (2)	N2—C13—C14—C10	58.07 (15)
O1—C1—C2—C3	176.38 (14)	C8—C10—C14—C13	176.30 (12)
C1—C2—C3—C4	0.9 (2)	C11—C10—C14—C13	−56.28 (15)
C2—C3—C4—C5	−179.87 (15)	C12—N2—C15—C16	176.08 (11)
C2—C3—C4—C9	1.8 (2)	C13—N2—C15—C16	−62.24 (15)
C9—C4—C5—C6	1.5 (2)	N2—C15—C16—C21	137.35 (13)
C3—C4—C5—C6	−176.80 (14)	N2—C15—C16—C17	−43.88 (18)
C4—C5—C6—C7	0.6 (2)	C21—C16—C17—C18	0.3 (2)
C5—C6—C7—C8	−1.3 (2)	C15—C16—C17—C18	−178.47 (14)
C6—C7—C8—C9	−0.1 (2)	C16—C17—C18—C19	−0.1 (2)
C6—C7—C8—C10	178.45 (13)	C17—C18—C19—C20	0.2 (2)
C1—N1—C9—C4	1.77 (19)	C18—C19—C20—C21	−0.5 (2)
C1—N1—C9—C8	−177.04 (12)	C18—C19—C20—C22	179.07 (14)
C5—C4—C9—N1	178.34 (13)	C17—C16—C21—C20	−0.7 (2)
C3—C4—C9—N1	−3.28 (19)	C15—C16—C21—C20	178.14 (13)
C5—C4—C9—C8	−2.9 (2)	C19—C20—C21—C16	0.8 (2)
C3—C4—C9—C8	175.50 (12)	C22—C20—C21—C16	−178.83 (13)
C7—C8—C9—N1	−178.98 (12)	C19—C20—C22—C26	−14.8 (2)
C10—C8—C9—N1	2.42 (18)	C21—C20—C22—C26	164.79 (15)
C7—C8—C9—C4	2.19 (18)	C19—C20—C22—C23	168.27 (15)
C10—C8—C9—C4	−176.41 (12)	C21—C20—C22—C23	−12.2 (2)
C7—C8—C10—C11	−27.88 (18)	C26—C22—C23—C24	6.9 (2)
C9—C8—C10—C11	150.65 (12)	C20—C22—C23—C24	−175.72 (14)
C7—C8—C10—C14	96.33 (16)	C22—C23—C24—C25	−12.1 (2)
C9—C8—C10—C14	−85.14 (15)	C23—C24—C25—C26	12.6 (2)
C8—C10—C11—C12	−177.56 (11)	C23—C22—C26—C25	1.4 (2)
C14—C10—C11—C12	55.98 (14)	C20—C22—C26—C25	−175.90 (16)
C15—N2—C12—C11	178.62 (11)	C24—C25—C26—C22	−9.0 (2)

### 8-{1-[3-(Cyclopent-1-en-1-yl)benzyl]piperidin-4-yl}-2-methoxyquinoline (I) Hydrogen-bond geometry (Å, º)

*CgB* is the centroid of ring C4–C9.

**Table:**

*D*—H···*A*	*D*—H	H···*A*	*D*···*A*	*D*—H···*A*
C18—H18···CgB^i^	0.95	2.97	3.661 (2)	131

Symmetry code: (i) *x*, *y*+1, *z*.

### 8-{4-[3-(Cyclopent-1-en-1-yl)benzyl]piperazin-1-yl}-2-methoxyquinoline (II) Crystal data

**Table:**

C_26_H_29_N_3_O	*Z* = 2
*M**_r_* = 399.52	*F*(000) = 428
Triclinic, *P*1	*D*_x_ = 1.226 Mg m^−^^3^
*a* = 7.9142 (8) Å	Mo *K*α radiation, λ = 0.71073 Å
*b* = 10.9051 (13) Å	Cell parameters from 5129 reflections
*c* = 12.8896 (14) Å	θ = 1.6–26.0°
α = 87.271 (9)°	µ = 0.08 mm^−^^1^
β = 79.290 (8)°	*T* = 173 K
γ = 82.206 (9)°	Plate, colourless
*V* = 1082.7 (2) Å^3^	0.45 × 0.40 × 0.19 mm

### 8-{4-[3-(Cyclopent-1-en-1-yl)benzyl]piperazin-1-yl}-2-methoxyquinoline (II) Data collection

**Table:**

STOE IPDS 2 diffractometer	4088 independent reflections
Radiation source: fine-focus sealed tube	2187 reflections with *I* > 2σ(*I*)
Plane graphite monochromator	*R*_int_ = 0.080
φ + ω scans	θ_max_ = 25.7°, θ_min_ = 1.6°
Absorption correction: multi-scan (MULABS; Spek, 2020)	*h* = −9→9
*T*_min_ = 0.793, *T*_max_ = 1.000	*k* = −13→13
11304 measured reflections	*l* = −15→15

### 8-{4-[3-(Cyclopent-1-en-1-yl)benzyl]piperazin-1-yl}-2-methoxyquinoline (II) Refinement

**Table:**

Refinement on *F*^2^	Secondary atom site location: difference Fourier map
Least-squares matrix: full	Hydrogen site location: inferred from neighbouring sites
*R*[*F*^2^ > 2σ(*F*^2^)] = 0.056	H-atom parameters constrained
*wR*(*F*^2^) = 0.121	*w* = 1/[σ^2^(*F*_o_^2^) + (0.048*P*)^2^] where *P* = (*F*_o_^2^ + 2*F*_c_^2^)/3
*S* = 0.90	(Δ/σ)_max_ < 0.001
4088 reflections	Δρ_max_ = 0.28 e Å^−^^3^
273 parameters	Δρ_min_ = −0.17 e Å^−^^3^
1 restraint	Extinction correction: (*SHELXL2018/3*; Sheldrick, 2015), Fc^*^=kFc[1+0.001xFc^2^λ^3^/sin(2θ)]^-1/4^
Primary atom site location: structure-invariant direct methods	Extinction coefficient: 0.0086 (15)

### 8-{4-[3-(Cyclopent-1-en-1-yl)benzyl]piperazin-1-yl}-2-methoxyquinoline (II) Special details

**Table:**

Geometry. All esds (except the esd in the dihedral angle between two l.s. planes) are estimated using the full covariance matrix. The cell esds are taken into account individually in the estimation of esds in distances, angles and torsion angles; correlations between esds in cell parameters are only used when they are defined by crystal symmetry. An approximate (isotropic) treatment of cell esds is used for estimating esds involving l.s. planes.

### 8-{4-[3-(Cyclopent-1-en-1-yl)benzyl]piperazin-1-yl}-2-methoxyquinoline (II) Fractional atomic coordinates and isotropic or equivalent isotropic displacement parameters (Å^2^)

**Table:**

	*x*	*y*	*z*	*U*_iso_*/*U*_eq_	
O1	−0.3136 (2)	−0.18899 (19)	0.38520 (16)	0.0549 (6)	
N1	−0.0986 (2)	−0.18024 (19)	0.23968 (17)	0.0372 (5)	
N2	0.2088 (2)	−0.09810 (17)	0.14688 (15)	0.0294 (5)	
N3	0.3825 (2)	0.10378 (18)	0.18985 (15)	0.0300 (5)	
C1	−0.2363 (3)	−0.2276 (2)	0.2863 (2)	0.0427 (7)	
C2	−0.3174 (3)	−0.3156 (3)	0.2433 (3)	0.0510 (8)	
H2	−0.416246	−0.347820	0.282598	0.061*	
C3	−0.2487 (3)	−0.3524 (3)	0.1439 (3)	0.0529 (8)	
H3	−0.300937	−0.410699	0.112140	0.063*	
C4	−0.0990 (3)	−0.3048 (2)	0.0861 (2)	0.0409 (7)	
C5	−0.0181 (4)	−0.3422 (2)	−0.0161 (2)	0.0476 (7)	
H5	−0.068608	−0.397363	−0.052471	0.057*	
C6	0.1322 (4)	−0.2996 (2)	−0.0632 (2)	0.0451 (7)	
H6	0.186715	−0.325569	−0.132045	0.054*	
C7	0.2067 (3)	−0.2173 (2)	−0.0102 (2)	0.0371 (6)	
H7	0.312281	−0.189311	−0.043911	0.045*	
C8	0.1314 (3)	−0.1759 (2)	0.08928 (19)	0.0318 (6)	
C9	−0.0260 (3)	−0.2196 (2)	0.1392 (2)	0.0349 (6)	
C10	0.3949 (3)	−0.0970 (2)	0.11133 (19)	0.0332 (6)	
H10A	0.416398	−0.059510	0.039396	0.040*	
H10B	0.454529	−0.182976	0.108390	0.040*	
C11	0.4666 (3)	−0.0238 (2)	0.1859 (2)	0.0343 (6)	
H11A	0.447282	−0.062345	0.257528	0.041*	
H11B	0.593116	−0.024947	0.161868	0.041*	
C12	0.1955 (3)	0.1034 (2)	0.22687 (19)	0.0326 (6)	
H12A	0.136438	0.189535	0.229207	0.039*	
H12B	0.175082	0.067128	0.299288	0.039*	
C13	0.1208 (3)	0.0294 (2)	0.1546 (2)	0.0348 (6)	
H13A	−0.004502	0.028411	0.181873	0.042*	
H13B	0.133604	0.069334	0.083420	0.042*	
C14	0.4552 (3)	0.1769 (2)	0.25913 (19)	0.0330 (6)	
H14A	0.583181	0.164805	0.239081	0.040*	
H14B	0.424670	0.146022	0.332804	0.040*	
C15	0.3914 (3)	0.3132 (2)	0.25410 (19)	0.0301 (6)	
C16	0.3790 (3)	0.3747 (2)	0.1577 (2)	0.0356 (6)	
H16	0.408753	0.329933	0.094036	0.043*	
C17	0.3238 (3)	0.5002 (2)	0.1546 (2)	0.0400 (7)	
H17	0.316755	0.541160	0.088555	0.048*	
C18	0.2787 (3)	0.5671 (2)	0.2464 (2)	0.0377 (6)	
H18	0.240296	0.653306	0.242779	0.045*	
C19	0.2891 (3)	0.5089 (2)	0.3440 (2)	0.0332 (6)	
C20	0.3453 (3)	0.3813 (2)	0.34630 (19)	0.0328 (6)	
H20	0.352236	0.340209	0.412330	0.039*	
C21	0.2444 (3)	0.5783 (2)	0.4432 (2)	0.0383 (6)	
C22	0.2269 (4)	0.5194 (3)	0.5478 (2)	0.0570 (8)	
H22A	0.339597	0.474384	0.559038	0.068*	
H22B	0.141230	0.459775	0.555661	0.068*	
C23	0.1659 (4)	0.6226 (3)	0.6270 (2)	0.0613 (9)	
H23A	0.043328	0.620057	0.660653	0.074*	
H23B	0.237817	0.614600	0.682791	0.074*	
C24	0.1860 (5)	0.7410 (3)	0.5641 (3)	0.0821 (11)	
H24A	0.283698	0.779644	0.580656	0.098*	
H24B	0.078692	0.800326	0.579663	0.098*	
C25	0.2211 (4)	0.7042 (3)	0.4504 (3)	0.0595 (8)	
H25	0.226301	0.760684	0.391774	0.071*	
C26	−0.2422 (4)	−0.0916 (3)	0.4257 (2)	0.0677 (10)	
H26C	−0.315015	−0.064309	0.492773	0.102*	
H26B	−0.237922	−0.021877	0.374866	0.102*	
H26A	−0.124641	−0.121897	0.437327	0.102*	

### 8-{4-[3-(Cyclopent-1-en-1-yl)benzyl]piperazin-1-yl}-2-methoxyquinoline (II) Atomic displacement parameters (Å^2^)

**Table:**

	*U*^11^	*U*^22^	*U*^33^	*U*^12^	*U*^13^	*U*^23^
O1	0.0422 (10)	0.0650 (15)	0.0550 (13)	−0.0133 (10)	−0.0015 (10)	0.0136 (11)
N1	0.0304 (11)	0.0367 (13)	0.0449 (14)	−0.0063 (9)	−0.0091 (10)	0.0088 (11)
N2	0.0300 (10)	0.0256 (11)	0.0329 (12)	−0.0053 (8)	−0.0051 (9)	−0.0015 (9)
N3	0.0299 (10)	0.0270 (11)	0.0339 (12)	−0.0045 (8)	−0.0062 (9)	−0.0046 (10)
C1	0.0364 (15)	0.0389 (17)	0.0523 (19)	−0.0057 (12)	−0.0106 (13)	0.0166 (15)
C2	0.0345 (15)	0.0446 (18)	0.078 (2)	−0.0162 (13)	−0.0175 (15)	0.0184 (17)
C3	0.0448 (16)	0.0356 (17)	0.087 (2)	−0.0114 (13)	−0.0326 (16)	0.0077 (17)
C4	0.0406 (15)	0.0266 (15)	0.061 (2)	−0.0037 (12)	−0.0267 (14)	0.0055 (14)
C5	0.0604 (18)	0.0298 (16)	0.061 (2)	−0.0014 (13)	−0.0353 (16)	−0.0038 (15)
C6	0.0587 (18)	0.0335 (16)	0.0455 (17)	0.0027 (13)	−0.0208 (14)	−0.0048 (14)
C7	0.0440 (14)	0.0301 (15)	0.0383 (16)	−0.0041 (11)	−0.0106 (12)	−0.0025 (13)
C8	0.0397 (14)	0.0217 (14)	0.0360 (15)	−0.0039 (11)	−0.0129 (12)	0.0018 (12)
C9	0.0367 (14)	0.0255 (14)	0.0439 (16)	−0.0004 (11)	−0.0156 (12)	0.0071 (13)
C10	0.0296 (13)	0.0285 (14)	0.0397 (15)	−0.0029 (10)	−0.0013 (11)	−0.0051 (12)
C11	0.0302 (13)	0.0325 (15)	0.0396 (16)	−0.0016 (11)	−0.0064 (11)	−0.0022 (12)
C12	0.0283 (12)	0.0285 (14)	0.0397 (15)	−0.0014 (10)	−0.0032 (11)	−0.0050 (12)
C13	0.0338 (13)	0.0267 (14)	0.0438 (16)	−0.0025 (11)	−0.0076 (12)	−0.0005 (12)
C14	0.0379 (13)	0.0317 (15)	0.0303 (14)	−0.0050 (11)	−0.0074 (11)	−0.0024 (12)
C15	0.0297 (12)	0.0262 (14)	0.0363 (15)	−0.0058 (10)	−0.0086 (11)	−0.0046 (12)
C16	0.0419 (14)	0.0340 (16)	0.0332 (15)	−0.0062 (11)	−0.0113 (12)	−0.0023 (13)
C17	0.0487 (16)	0.0367 (16)	0.0388 (16)	−0.0092 (13)	−0.0184 (13)	0.0087 (13)
C18	0.0388 (14)	0.0277 (15)	0.0500 (17)	−0.0053 (11)	−0.0159 (13)	−0.0008 (14)
C19	0.0266 (12)	0.0329 (15)	0.0418 (16)	−0.0071 (10)	−0.0073 (11)	−0.0070 (13)
C20	0.0339 (13)	0.0350 (15)	0.0319 (15)	−0.0091 (11)	−0.0095 (11)	0.0009 (12)
C21	0.0369 (14)	0.0375 (16)	0.0418 (17)	−0.0018 (11)	−0.0113 (12)	−0.0086 (13)
C22	0.076 (2)	0.051 (2)	0.0433 (18)	0.0013 (16)	−0.0137 (15)	−0.0124 (16)
C23	0.068 (2)	0.063 (2)	0.0516 (19)	0.0022 (16)	−0.0085 (16)	−0.0237 (18)
C24	0.113 (3)	0.054 (2)	0.077 (3)	0.010 (2)	−0.018 (2)	−0.029 (2)
C25	0.076 (2)	0.0426 (19)	0.054 (2)	0.0021 (16)	−0.0013 (16)	−0.0145 (16)
C26	0.063 (2)	0.087 (3)	0.051 (2)	−0.0196 (19)	0.0029 (16)	−0.0046 (19)

### 8-{4-[3-(Cyclopent-1-en-1-yl)benzyl]piperazin-1-yl}-2-methoxyquinoline (II) Geometric parameters (Å, º)

**Table:**

O1—C1	1.367 (3)	C13—H13A	0.9900
O1—C26	1.430 (4)	C13—H13B	0.9900
N1—C1	1.302 (3)	C14—C15	1.506 (3)
N1—C9	1.378 (3)	C14—H14A	0.9900
N2—C8	1.420 (3)	C14—H14B	0.9900
N2—C10	1.460 (3)	C15—C20	1.395 (3)
N2—C13	1.468 (3)	C15—C16	1.397 (3)
N3—C11	1.457 (3)	C16—C17	1.380 (3)
N3—C14	1.466 (3)	C16—H16	0.9500
N3—C12	1.468 (3)	C17—C18	1.383 (3)
C1—C2	1.410 (4)	C17—H17	0.9500
C2—C3	1.352 (4)	C18—C19	1.394 (3)
C2—H2	0.9500	C18—H18	0.9500
C3—C4	1.424 (4)	C19—C20	1.403 (3)
C3—H3	0.9500	C19—C21	1.477 (3)
C4—C5	1.407 (4)	C20—H20	0.9500
C4—C9	1.421 (3)	C21—C25	1.365 (4)
C5—C6	1.361 (4)	C21—C22	1.457 (4)
C5—H5	0.9500	C22—C23	1.524 (4)
C6—C7	1.404 (3)	C22—H22A	0.9900
C6—H6	0.9500	C22—H22B	0.9900
C7—C8	1.379 (3)	C23—C24	1.502 (5)
C7—H7	0.9500	C23—H23A	0.9900
C8—C9	1.423 (3)	C23—H23B	0.9900
C10—C11	1.511 (3)	C24—C25	1.504 (4)
C10—H10A	0.9900	C24—H24A	0.9900
C10—H10B	0.9900	C24—H24B	0.9900
C11—H11A	0.9900	C25—H25	0.9500
C11—H11B	0.9900	C26—H26C	0.9800
C12—C13	1.508 (3)	C26—H26B	0.9800
C12—H12A	0.9900	C26—H26A	0.9800
C12—H12B	0.9900		
			
C1—O1—C26	116.1 (2)	C12—C13—H13B	109.4
C1—N1—C9	117.0 (2)	H13A—C13—H13B	108.0
C8—N2—C10	114.96 (19)	N3—C14—C15	113.2 (2)
C8—N2—C13	113.51 (18)	N3—C14—H14A	108.9
C10—N2—C13	109.68 (18)	C15—C14—H14A	108.9
C11—N3—C14	110.95 (18)	N3—C14—H14B	108.9
C11—N3—C12	108.63 (18)	C15—C14—H14B	108.9
C14—N3—C12	110.84 (18)	H14A—C14—H14B	107.8
N1—C1—O1	118.5 (2)	C20—C15—C16	118.5 (2)
N1—C1—C2	125.8 (3)	C20—C15—C14	120.3 (2)
O1—C1—C2	115.6 (2)	C16—C15—C14	121.2 (2)
C3—C2—C1	117.3 (3)	C17—C16—C15	120.3 (2)
C3—C2—H2	121.3	C17—C16—H16	119.9
C1—C2—H2	121.3	C15—C16—H16	119.9
C2—C3—C4	120.9 (3)	C16—C17—C18	120.9 (3)
C2—C3—H3	119.5	C16—C17—H17	119.6
C4—C3—H3	119.5	C18—C17—H17	119.6
C5—C4—C9	119.8 (2)	C17—C18—C19	120.5 (2)
C5—C4—C3	123.6 (3)	C17—C18—H18	119.8
C9—C4—C3	116.5 (3)	C19—C18—H18	119.8
C6—C5—C4	120.2 (3)	C18—C19—C20	118.1 (2)
C6—C5—H5	119.9	C18—C19—C21	121.7 (2)
C4—C5—H5	119.9	C20—C19—C21	120.2 (2)
C5—C6—C7	120.1 (3)	C15—C20—C19	121.7 (2)
C5—C6—H6	119.9	C15—C20—H20	119.1
C7—C6—H6	119.9	C19—C20—H20	119.1
C8—C7—C6	122.1 (2)	C25—C21—C22	110.7 (3)
C8—C7—H7	119.0	C25—C21—C19	125.7 (3)
C6—C7—H7	119.0	C22—C21—C19	123.6 (2)
C7—C8—N2	123.2 (2)	C21—C22—C23	106.6 (3)
C7—C8—C9	118.3 (2)	C21—C22—H22A	110.4
N2—C8—C9	118.4 (2)	C23—C22—H22A	110.4
N1—C9—C4	122.4 (2)	C21—C22—H22B	110.4
N1—C9—C8	118.2 (2)	C23—C22—H22B	110.4
C4—C9—C8	119.4 (2)	H22A—C22—H22B	108.6
N2—C10—C11	110.24 (19)	C24—C23—C22	105.4 (3)
N2—C10—H10A	109.6	C24—C23—H23A	110.7
C11—C10—H10A	109.6	C22—C23—H23A	110.7
N2—C10—H10B	109.6	C24—C23—H23B	110.7
C11—C10—H10B	109.6	C22—C23—H23B	110.7
H10A—C10—H10B	108.1	H23A—C23—H23B	108.8
N3—C11—C10	110.41 (19)	C23—C24—C25	105.3 (3)
N3—C11—H11A	109.6	C23—C24—H24A	110.7
C10—C11—H11A	109.6	C25—C24—H24A	110.7
N3—C11—H11B	109.6	C23—C24—H24B	110.7
C10—C11—H11B	109.6	C25—C24—H24B	110.7
H11A—C11—H11B	108.1	H24A—C24—H24B	108.8
N3—C12—C13	110.67 (19)	C21—C25—C24	110.5 (3)
N3—C12—H12A	109.5	C21—C25—H25	124.8
C13—C12—H12A	109.5	C24—C25—H25	124.8
N3—C12—H12B	109.5	O1—C26—H26C	109.5
C13—C12—H12B	109.5	O1—C26—H26B	109.5
H12A—C12—H12B	108.1	H26C—C26—H26B	109.5
N2—C13—C12	111.01 (19)	O1—C26—H26A	109.5
N2—C13—H13A	109.4	H26C—C26—H26A	109.5
C12—C13—H13A	109.4	H26B—C26—H26A	109.5
N2—C13—H13B	109.4		
			
C9—N1—C1—O1	−178.8 (2)	C12—N3—C11—C10	59.9 (2)
C9—N1—C1—C2	0.1 (4)	N2—C10—C11—N3	−60.3 (3)
C26—O1—C1—N1	4.6 (3)	C11—N3—C12—C13	−58.6 (2)
C26—O1—C1—C2	−174.5 (2)	C14—N3—C12—C13	179.3 (2)
N1—C1—C2—C3	−1.6 (4)	C8—N2—C13—C12	173.4 (2)
O1—C1—C2—C3	177.4 (2)	C10—N2—C13—C12	−56.5 (3)
C1—C2—C3—C4	0.8 (4)	N3—C12—C13—N2	57.6 (3)
C2—C3—C4—C5	177.9 (3)	C11—N3—C14—C15	171.8 (2)
C2—C3—C4—C9	1.1 (4)	C12—N3—C14—C15	−67.4 (3)
C9—C4—C5—C6	1.7 (4)	N3—C14—C15—C20	137.7 (2)
C3—C4—C5—C6	−175.0 (3)	N3—C14—C15—C16	−43.2 (3)
C4—C5—C6—C7	−0.5 (4)	C20—C15—C16—C17	0.6 (3)
C5—C6—C7—C8	−0.7 (4)	C14—C15—C16—C17	−178.6 (2)
C6—C7—C8—N2	176.5 (2)	C15—C16—C17—C18	−0.5 (4)
C6—C7—C8—C9	0.6 (4)	C16—C17—C18—C19	0.4 (3)
C10—N2—C8—C7	−19.3 (3)	C17—C18—C19—C20	−0.4 (3)
C13—N2—C8—C7	108.1 (3)	C17—C18—C19—C21	179.0 (2)
C10—N2—C8—C9	156.7 (2)	C16—C15—C20—C19	−0.6 (3)
C13—N2—C8—C9	−75.9 (3)	C14—C15—C20—C19	178.6 (2)
C1—N1—C9—C4	2.0 (3)	C18—C19—C20—C15	0.5 (3)
C1—N1—C9—C8	−175.7 (2)	C21—C19—C20—C15	−178.9 (2)
C5—C4—C9—N1	−179.5 (2)	C18—C19—C21—C25	−12.9 (4)
C3—C4—C9—N1	−2.6 (3)	C20—C19—C21—C25	166.5 (3)
C5—C4—C9—C8	−1.9 (3)	C18—C19—C21—C22	170.0 (2)
C3—C4—C9—C8	175.1 (2)	C20—C19—C21—C22	−10.7 (3)
C7—C8—C9—N1	178.5 (2)	C25—C21—C22—C23	7.0 (3)
N2—C8—C9—N1	2.4 (3)	C19—C21—C22—C23	−175.5 (2)
C7—C8—C9—C4	0.7 (3)	C21—C22—C23—C24	−11.4 (3)
N2—C8—C9—C4	−175.4 (2)	C22—C23—C24—C25	11.3 (4)
C8—N2—C10—C11	−173.2 (2)	C22—C21—C25—C24	0.3 (4)
C13—N2—C10—C11	57.5 (2)	C19—C21—C25—C24	−177.1 (3)
C14—N3—C11—C10	−178.03 (19)	C23—C24—C25—C21	−7.6 (4)

### 8-{4-[3-(Cyclopent-1-en-1-yl)benzyl]piperazin-1-yl}-2-methoxyquinoline (II) Hydrogen-bond geometry (Å, º)

*CgB* is the centroid of ring C4-C9.

**Table:**

*D*—H···*A*	*D*—H	H···*A*	*D*···*A*	*D*—H···*A*
C13—H13*B*···CgB^i^	0.99	2.95	3.757 (3)	140
C17—H17···CgB^ii^	0.95	2.93	3.602 (3)	129

Symmetry codes: (i) −*x*, −*y*, −*z*; (ii) *x*, *y*+1, *z*.
