# Supplementary material for: The crystal structures, Hirshfeld surface analyses and energy frameworks of 8-{1-[3-(cyclo­pent-1-en-1-yl)benz­yl]piperidin-4-yl}-2-meth­oxy­quinoline and 8-{4-[3-(cyclo­pent-1-en-1-yl)benz­yl]piperazin-1-yl}-2-meth­oxy­quinoline
Source: Acta Crystallogr E Crystallogr Commun. 2021 Mar 9;77(Pt 4):372–7. doi: 10.1107/S2056989021002474 (PMC8025868; doi:10.1107/S2056989021002474)
Supplement: Supplementary file 6 [file e-77-00372-sup6.pdf]

Data Analysis

File Options

search2 Spreadsheet 1

File Tools Descriptors Display Selection Plots Statistics

Find identifier | Find next

Identifier	NAME	...ery	...gment	...ST1	...ST2	...ST3	...ST4	...ST5
search2|AKUWOV|0	AKUWOV	1	1	1.3590	1.5120	1.5200	1.5510	1.4850
search2|AKUWOV|1	AKUWOV	1	2	1.3610	1.4760	1.5020	1.5480	1.4800
search2|AKUWUB|2	AKUWUB	1	1	1.3360	1.4980	1.5460	1.5130	1.4940
search2|ANONUO|3	ANONUO	1	1	1.3420	1.4940	1.5320	1.5110	1.4890
search2|AXEPUP|4	AXEPUP	1	1	1.3260	1.5100	1.5510	1.5380	1.5030
search2|BECNUV|5	BECNUV	1	1	1.3380	1.5100	1.5440	1.5320	1.4960
search2|BECNUV|6	BECNUV	1	2	1.3440	1.5010	1.5400	1.5320	1.5020
search2|CAZHAO|7	CAZHAO	1	1	1.3180	1.5090	1.5030	1.5030	1.5080
search2|CIZLOM|8	CIZLOM	1	1	1.3190	1.5030	1.4970	1.4900	1.4940
search2|CPCBCR|9	CPCBCR	1	1	1.3460	1.5300	1.5290	1.4770	1.5640
search2|CUHPAX|10	CUHPAX	1	1	1.3640	1.4200	1.4960	1.4790	1.4900
search2|DOLZIN|11	DOLZIN	1	1	1.3720	1.4430	1.5260	1.4950	1.5280
search2|EFELAF|12	EFELAF	1	1	1.3960	1.3910	1.5070	1.4770	1.4950
search2|FAXDIT|13	FAXDIT	1	1	1.3370	1.5110	1.5390	1.5310	1.4870
search2|HOBYUT|14	HOBYUT	1	1	1.3290	1.4970	1.5230	1.5140	1.4780
search2|IVIXOA|15	IVIXOA	1	1	1.3210	1.5130	1.5330	1.5260	1.5040
search2|IVIXOA|16	IVIXOA	1	2	1.3290	1.5110	1.5100	1.4630	1.5130
search2|IVIXOA|17	IVIXOA	1	3	1.3740	1.4380	1.5270	1.4650	1.4980
search2|IVIXOA|18	IVIXOA	1	4	1.3820	1.4400	1.5170	1.5150	1.5020
search2|IVIXUG|19	IVIXUG	1	1	1.3260	1.4280	1.5170	1.4850	1.4970
search2|IVIXUG|20	IVIXUG	1	2	1.3480	1.4610	1.5360	1.4940	1.4980

search2 Descriptive Statistics 1

File Descriptors Display Selection Plots Statistics

	Name	Count	Missing	Selected	Minimum	Maximum	Sum	Mean	Variance	Std. Dev	Mean. Dev	Skewness	Kurtosis	Median	Lo
1	DIST1	50	0	0	1,268	1,417	67,39	1.348	0.001	0.029	0.024	0.311	-0.066	1.340	
2	DIST2	50	0	0	1,391	1,534	74.036	1.481	0.001	0.034	0.029	-0.646	-0.720	1.496	

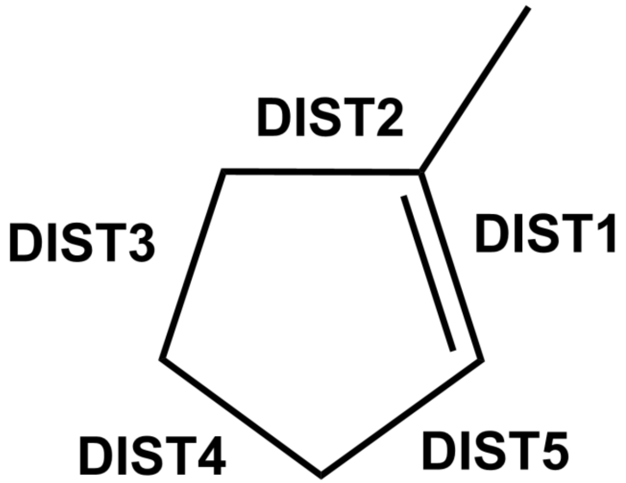

Search Overview

Search:	search2
Date/Time done:	Thu Mar 4 14:53:55 2021
Database(s):	CSD version 5.42 updates (Feb 2021)<br>CSD version 5.42 (November 2020)
Restriction Info:	No refcode restrictions applied
Filters:	None
Percentage Completed:	100%
Number of Hits:	40

Single query used. Search found structures that:

match

Query 1

Query 1

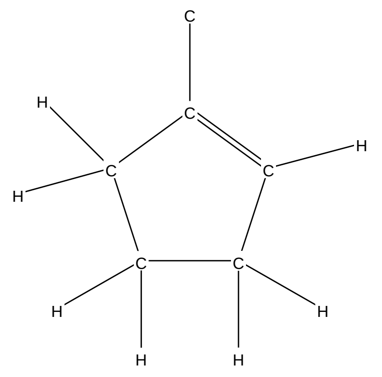

Search: search2 (Thu Mar 4 14:53:55 2021): Hits 1-4

AKUWOV

Reference: N.Ullah, M.Altaf, M.Mansha, A.O.Ba-Salem (2015) *J.Struct.Chem.* ,**56,**1441

Formula: $C_{25} H_{29} N_3 O_1$

Compound Name: 8-(1-{[5-(cyclopent-1-en-1-yl)pyridin-3-yl]methyl}piperidin-4-yl)-3,4-dihydroquinolin-2(1H)-one

Space Group:	P21/n	***Cell:***	***a***	17.739(2)	***b***	9.075(0)	***c***	26.440(3)
Space Group No.:	14	***(Å,°)***	α	90.00	β	106.51(0)	γ	90.00

R-Factor (%): 3.76 ***Temperature(K):*** 173 ***Density(g/cm^3):*** 1.262

Parameters

Fragment 1

**DIST1 (D)**	1.359
**DIST2 (D)**	1.512
**DIST3 (D)**	1.520
**DIST4 (D)**	1.551
**DIST5 (D)**	1.485

Fragment 2

**DIST1 (D)**	1.361
**DIST2 (D)**	1.476
**DIST3 (D)**	1.502
**DIST4 (D)**	1.548
**DIST5 (D)**	1.480

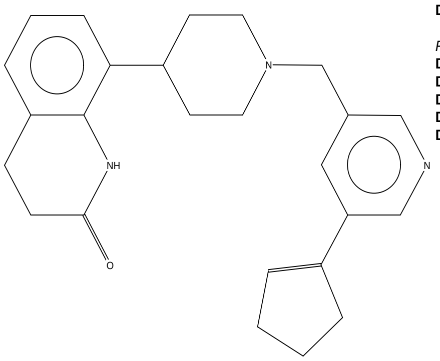

AKUWUB

Reference: N.Ullah, M.Altaf, M.Mansha, A.O.Ba-Salem (2015) *J.Struct.Chem.* ,**56,**1441

Formula: $C_{26} H_{30} N_2 O_1$

Compound Name: 8-(1-{[3-(cyclopent-1-en-1-yl)phenyl]methyl}piperidin-4-yl)-3,4-dihydroquinolin-2(1H)-one

Space Group:	P-1	***Cell:***	***a***	7.410(1)	***b***	12.372(3)	***c***	13.441(4)
Space Group No.:	2	***(Å,°)***	α	63.95(1)	β	85.56(2)	γ	77.68(1)

R-Factor (%): 6.84 ***Temperature(K):*** 173 ***Density(g/cm^3):*** 1.187

Parameters

Fragment 1

**DIST1 (D)**	1.336
**DIST2 (D)**	1.498
**DIST3 (D)**	1.546
**DIST4 (D)**	1.513
**DIST5 (D)**	1.494

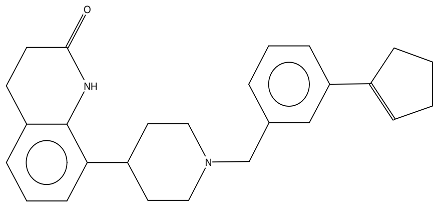

ANONUO

Reference: T.Saito, Y.Sonoki, T.Otani, N.Kutsumura (2014) *Org.Biomol.Chem.* ,**12,**8398

Formula: $C_{14} H_{13} N_1 O_1$

Compound Name: 3-(cyclopent-1-en-1-ylmethylene)-1,3-dihydro-2H-indol-2-one

Space Group:	P21/c	***Cell:***	***a***	10.813(1)	***b***	6.502(1)	***c***	16.577(3)
Space Group No.:	14	***(Å,°)***	α	90.00	β	105.35(0)	γ	90.00

R-Factor (%): 6.59 ***Temperature(K):*** 291 ***Density(g/cm^3):*** 1.248

Parameters

Fragment 1

**DIST1 (D)**	1.342
**DIST2 (D)**	1.494
**DIST3 (D)**	1.532
**DIST4 (D)**	1.511
**DIST5 (D)**	1.489

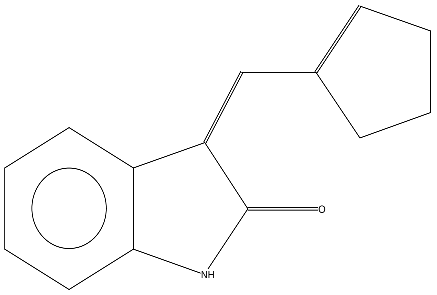

AXEPUP

Reference: Yajing Lian, H.M.L.Davies (2011) *J.Am.Chem.Soc.* ,**133,** 11940

Formula: $C_{22} H_{24} O_3$

Compound Name: Methyl 5-(cyclopent-1-en-1-yl)-5-methoxy-4-(2-naphthyl)pent-2-enoate

Space Group:	P21	***Cell:***	***a***	8.853(0)	***b***	6.232(0)	***c***	16.867(1)
Space Group No.:	4	***(Å,°)***	α	90.00	β	102.24(0)	γ	90.00

R-Factor (%): 3.83 ***Temperature(K):*** 173 ***Density(g/cm^3):*** 1.229

Parameters

Fragment 1

**DIST1 (D)**	1.326
**DIST2 (D)**	1.510
**DIST3 (D)**	1.551
**DIST4 (D)**	1.538
**DIST5 (D)**	1.503

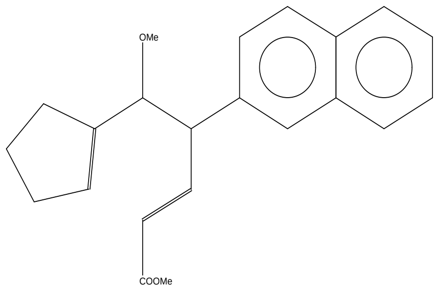

Search: search2 (Thu Mar 4 14:53:55 2021): Hits 5-8

BECNUV

Reference: B.W.Skelton, P.A.Keller (2017)
CSD Communication(Private Communication) ,

Formula: $C_{12} H_{12} I_1 N_1 O_1$

Compound Name: N-(2-iodophenyl)cyclopent-1-ene-1-carboxamide

Space Group:	P-1	***Cell:***	***a*** 7.605(0)	***b*** 11.856(0)	***c*** 12.819(0)
Space Group No.:	2	***(Å,°)***	α 88.13(0)	β 85.25(0)	γ 87.59(0)

R-Factor (%): 2.58 ***Temperature(K):*** 150 ***Density(g/cm^3):*** 1.808

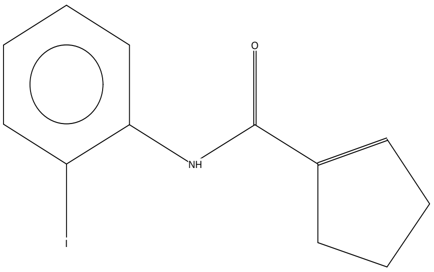

Parameters

Fragment 1	
**DIST1 (D)**	1.338
**DIST2 (D)**	1.510
**DIST3 (D)**	1.544
**DIST4 (D)**	1.532
**DIST5 (D)**	1.496

Fragment 2	
**DIST1 (D)**	1.344
**DIST2 (D)**	1.501
**DIST3 (D)**	1.540
**DIST4 (D)**	1.532
**DIST5 (D)**	1.502

CAZHAO

Reference: Kai-Ming Guo, Zhi-Hong Ma, Su-Zhen Li, Zhan-Gang Han, Jin Lin, Xue-Zhong Zheng (2012)
Wuji Huaxue Xuebao(Chin.)(Chin.J.Inorg.Chem.) ,**28,**643

Formula: $C_{32} H_{26} O_4 Ru_2$

Compound Name: bis(μ_2-carbonyl)-bis(η^5-1-(cyclopent-1-en-1-yl)indenyl)-dicarbonyl-di-ruthenium

Space Group:	P21/c	***Cell:***	***a*** 7.571(1)	***b*** 15.777(3)	***c*** 11.073(1)
Space Group No.:	14	***(Å,°)***	α 90.00	β 90.07(2)	γ 90.00

R-Factor (%): 3.05 ***Temperature(K):*** 298 ***Density(g/cm^3):*** 1.699

Parameters

Fragment 1	
**DIST1 (D)**	1.318
**DIST2 (D)**	1.509
**DIST3 (D)**	1.503
**DIST4 (D)**	1.503
**DIST5 (D)**	1.508

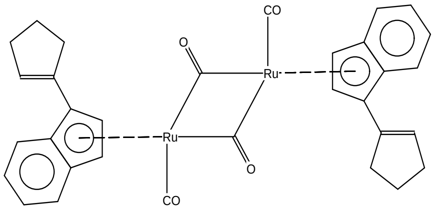

CIZLOM

Reference: J.C.Caille, M.Farnier, R.Guilard, A.Aubry, C.Lecomte
(1986) *Can.J.Chem.* ,**64,**831

Formula: $C_{13} H_{16} O_2$

Compound Name: 4a-Cyclopentenyl-1,3,4,5,6-pentahydrocyclopenta(c)pyran-1-one

Space Group:	I2/c	***Cell:***	***a*** 17.144(7)	***b*** 6.274(3)	***c*** 20.977(7)
Space Group No.:	15	***(Å,°)***	α 90.00	β 98.66(3)	γ 90.00

R-Factor (%): 5.10 ***Temperature(K):*** 295 ***Density(g/cm^3):*** 1.217

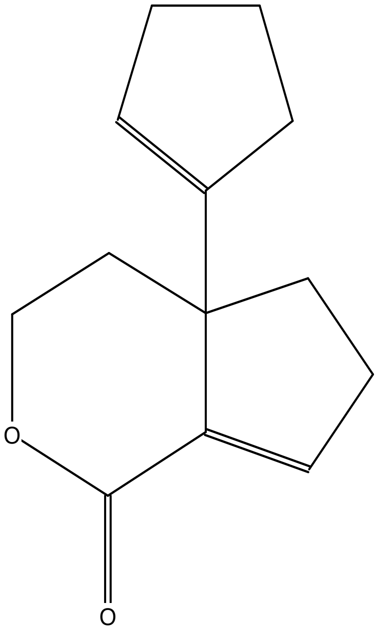

Parameters

Fragment 1	
**DIST1 (D)**	1.319
**DIST2 (D)**	1.503
**DIST3 (D)**	1.497
**DIST4 (D)**	1.490
**DIST5 (D)**	1.494

CPCBCR

Reference: E.O.Fischer, W.R.Wagner, F.R.Kreissl, D.Neugebauer
(1979) *Chem.Ber.* ,**112,**1320

Formula: $C_{10} H_7 Cr_1 I_1 O_4, C_1 H_2 Cl_2$

Compound Name: trans-Tetracarbonyl-((1-cyclopentenyl)carbyne)-iodo-chromium dichloromethane solvate

Space Group:	P212121	***Cell:***	***a*** 10.070(20)	***b*** 12.430(20)	***c*** 13.150(20)
Space Group No.:	19	***(Å,°)***	α 90.00	β 90.00	γ 90.00

R-Factor (%): 5.20 ***Temperature(K):*** 233 ***Density(g/cm^3):*** 1.836

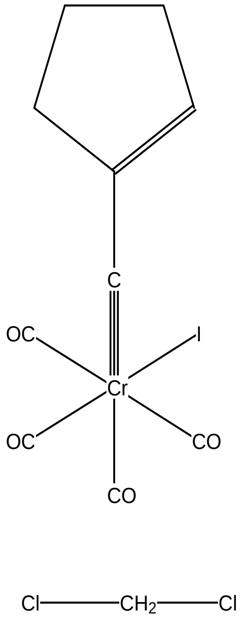

Parameters

Fragment 1	
**DIST1 (D)**	1.346
**DIST2 (D)**	1.530
**DIST3 (D)**	1.529
**DIST4 (D)**	1.477
**DIST5 (D)**	1.564

Search: search2 (Thu Mar 4 14:53:55 2021): Hits 9-12

CUHPAX

Reference: Kung-Pern Wang, Sang Young Yun, Daesung Lee, D.J.Wink (2009) *J.Am.Chem.Soc.* ,**131,**15114

Formula: $C_{37}\ H_{46}\ Cl_2\ N_2\ Ru_1$

Compound Name: (η^2-(2-(Cyclopent-1-en-1-ylethynyl)-6,6-dimethylcyclohex-1-en-1-yl) methylene)-dichloro-(1,3-dimesitylimidazolidin-2-ylidene)-ruthenium

Space Group:	P21	***Cell:***	***a*** 12.015(1)	***b*** 10.651(1)	***c*** 13.598(1)
Space Group No.:	4	***(Å,°)***	α 90.00	β 94.06(0)	γ 90.00

R-Factor (%): 3.79 ***Temperature(K):*** 298 ***Density(g/cm^3):*** 1.322

Parameters

Fragment 1	
**DIST1 (D)**	1.364
**DIST2 (D)**	1.420
**DIST3 (D)**	1.496
**DIST4 (D)**	1.479
**DIST5 (D)**	1.490

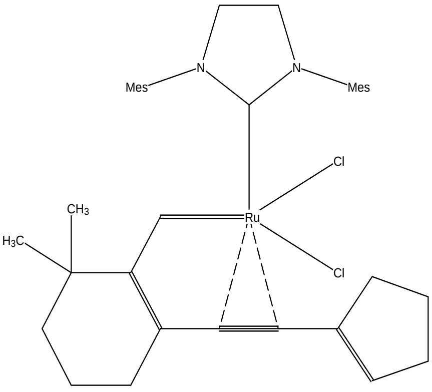

DOLZIN

Reference: R.Aumann, R.Frohlich, J.Prigge, O.Meyer (1999) *Organometallics* ,**18,**1369

Formula: $C_{17}\ H_{19}\ N_1\ O_6\ W_1$

Compound Name: (3E)-4-(Cyclopentenyl)-4-(dimethylamino)-2-ethoxy-1,1,1,1,1-pentacarbonyl-1-tungsta-1,3-butadiene

Synonym: Pentacarbonyl-(3-cyclopentenyl-1-ethoxy-3-dimethylaminoprop-2-enylidene)-tungsten

Space Group:	P-1	***Cell:***	***a*** 9.413(1)	***b*** 10.337(1)	***c*** 11.643(1)
Space Group No.:	2	***(Å,°)***	α 112.01(1)	β 105.69(1)	γ 102.42(1)

R-Factor (%): 3.87 ***Temperature(K):*** 223 ***Density(g/cm^3):*** 1.817

Parameters

Fragment 1	
**DIST1 (D)**	1.372
**DIST2 (D)**	1.443
**DIST3 (D)**	1.526
**DIST4 (D)**	1.495
**DIST5 (D)**	1.528

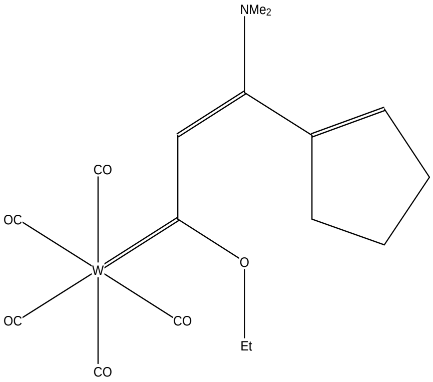

EFELAF

Reference: Yulong Song, Wangteng Wu, Chunling Fu, Xin Huang, Shengming Ma (2019) *Adv.Synth.Catal.* ,**361,**3228

Formula: $C_{30}\ H_{30}\ N_2\ O_6\ S_2, C_1\ H_1\ Cl_3$

Compound Name: N-({4-(cyclopent-1-en-1-yl)-7-[(4-methylphenyl)sulfonyl]-8-oxo-3,6,7,8-tetrahydro-1H-furo[3,4-e]isoindol-5-yl}methyl)-4-methylbenzene-1-sulfonamide chloroform solvate

Space Group:	P-1	***Cell:***	***a*** 6.894(0)	***b*** 15.717(0)	***c*** 16.680(0)
Space Group No.:	2	***(Å,°)***	α 110.05(0)	β 95.58(0)	γ 98.67(0)

R-Factor (%): 7.29 ***Temperature(K):*** 293 ***Density(g/cm^3):*** 1.399

Parameters

Fragment 1	
**DIST1 (D)**	1.396
**DIST2 (D)**	1.391
**DIST3 (D)**	1.507
**DIST4 (D)**	1.477
**DIST5 (D)**	1.495

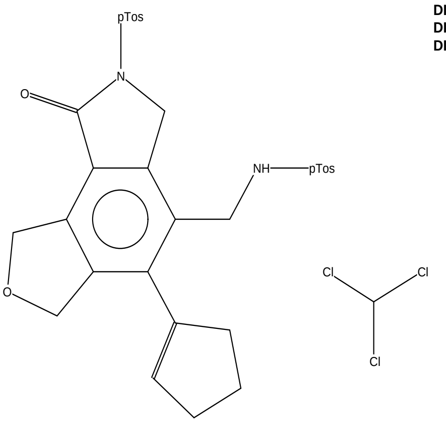

FAXDIT

Reference: A.S.K.Hashmi, I.Braun, P.Nosel, J.Schadlich, M.Wieteck, M.Rudolph, F.Rominger (2012) *Angew.Chem.,Int.Ed.* ,**51,**4456

Formula: $C_{15}\ H_{14}$

Compound Name: 1-(Cyclopent-1-en-1-ylmethylene)-1H-indene

Space Group:	P21/n	***Cell:***	***a*** 9.138(0)	***b*** 5.699(0)	***c*** 20.283(1)
Space Group No.:	14	***(Å,°)***	α 90.00	β 95.08(0)	γ 90.00

R-Factor (%): 4.48 ***Temperature(K):*** 200 ***Density(g/cm^3):*** 1.226

Parameters

Fragment 1	
**DIST1 (D)**	1.337
**DIST2 (D)**	1.511
**DIST3 (D)**	1.539
**DIST4 (D)**	1.531
**DIST5 (D)**	1.487

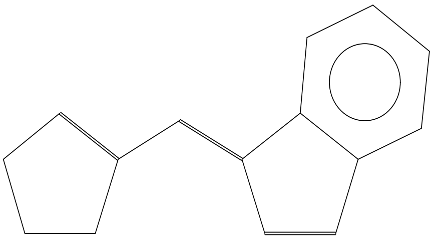

HOBYUT

Reference: Yu-min Liu, He Liu, Yong-qi Hu (2008) *J.Chem.Cryst.* ,**38,** 491

Formula: $C_{20}\,H_{25}\,N_1\,O_3$

Compound Name: 3'-Quinuclidinyl 2-cyclopentenyl-2-hydroxy-2-phenylacetate

Space Group:	P212121	***Cell:***	***a***	8.547(2)	***b***	11.428(4)	***c***	18.288(5)
Space Group No.:	19	***(Å,°)***	α	90.00	β	90.00	γ	90.00

R-Factor (%): 5.18 ***Temperature(K):*** 293 ***Density(g/cm^3):*** 1.218

Parameters

Fragment 1	
**DIST1 (D)**	1.329
**DIST2 (D)**	1.497
**DIST3 (D)**	1.523
**DIST4 (D)**	1.514
**DIST5 (D)**	1.478

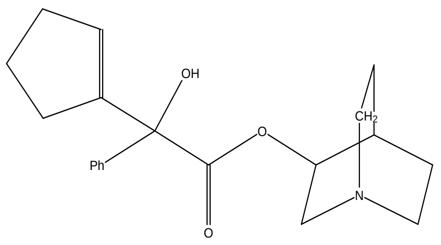

IVIXOA

Reference: V.B.Golovko, M.J.Mays, A.D.Woods (2002) *New J.Chem.* , **26,**1706

Formula: $C_{43}\,H_{36}\,Co_2\,O_4\,P_2$

Compound Name: (μ_2-bis(Diphenylphosphino)methane)-(μ_2-η^2,η^2-1,4-dicyclopentenylbuta-1,3-diyne)-tetracarbonyl-di-cobalt

Space Group:	P-1	***Cell:***	***a***	12.276(0)	***b***	15.309(0)	***c***	21.746(0)
Space Group No.:	2	***(Å,°)***	α	104.38(0)	β	90.55(0)	γ	107.00(0)

R-Factor (%): 4.95 ***Temperature(K):*** 180 ***Density(g/cm^3):*** 1.403

Parameters

	Fragment 1	Fragment 2	Fragment 3	Fragment 4
**DIST1 (D)**	1.321	1.329	1.374	1.382
**DIST2 (D)**	1.513	1.511	1.438	1.440
**DIST3 (D)**	1.533	1.510	1.527	1.517
**DIST4 (D)**	1.526	1.463	1.465	1.515
**DIST5 (D)**	1.504	1.513	1.498	1.502

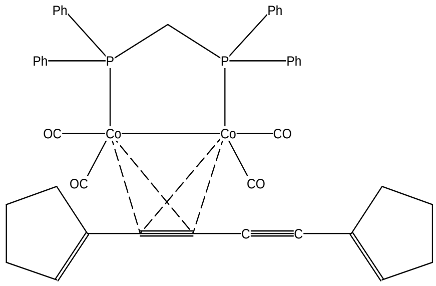

IVIXUG

Reference: V.B.Golovko, M.J.Mays, A.D.Woods (2002) *New J.Chem.* , **26,**1706

Formula: $C_{26}\,H_{14}\,Co_4\,O_{12}$

Compound Name: (μ_4-η^2,η^2,η^2,η^2-1,4-Dicyclopentenylbuta-1,3-diyne)-dodecacarbonyl-tetra-cobalt

Space Group:	P-1	***Cell:***	***a***	11.119(0)	***b***	13.790(0)	***c***	18.772(0)
Space Group No.:	2	***(Å,°)***	α	91.38(0)	β	92.81(0)	γ	97.59(0)

R-Factor (%): 4.42 ***Temperature(K):*** 180 ***Density(g/cm^3):*** 1.759

Parameters

	Fragment 1	Fragment 2	Fragment 3	Fragment 4
**DIST1 (D)**	1.326	1.348	1.356	1.325
**DIST2 (D)**	1.428	1.461	1.477	1.492
**DIST3 (D)**	1.517	1.536	1.515	1.521
**DIST4 (D)**	1.485	1.494	1.508	1.503
**DIST5 (D)**	1.497	1.498	1.512	1.501

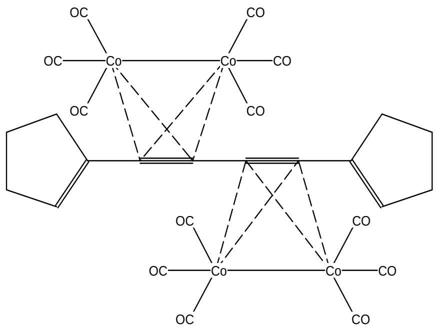

JOFWEH

Reference: Nu Xiao, Jie Sun, Huping Zhu, N.Tsumori, Jiabi Chen (2008) *Inorg.Chim.Acta* ,**361,**3171

Formula: $C_{39}\,H_{39}\,Fe_1\,O_3\,P_1$,$C_4\,H_{10}\,O_1$

Compound Name: (η^4-1-(Cyclopentenyl)-5-ethoxy-5-o-tolyl-1,4-pentadiene)-dicarbonyl-(triphenylphosphine)-iron diethyl ether solvate

Space Group:	C2/c	***Cell:***	***a***	36.861(3)	***b***	13.757(1)	***c***	14.856(1)
Space Group No.:	15	***(Å,°)***	α	90.00	β	106.07(0)	γ	90.00

R-Factor (%): 5.19 ***Temperature(K):*** 293 ***Density(g/cm^3):*** 1.315

Parameters

Fragment 1	
**DIST1 (D)**	1.407
**DIST2 (D)**	1.504
**DIST3 (D)**	1.523
**DIST4 (D)**	1.527
**DIST5 (D)**	1.515

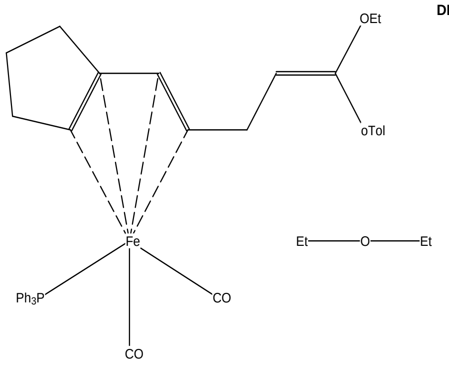

Search: search2 (Thu Mar 4 14:53:55 2021): Hits 17-20

KAMKOA

Reference: Li-Juan Tian, Zhi-Hong Ma, Zhan-Gang Han, Xue-Zhong Zheng, Jin Lin (2011) *Transition Met.Chem.* ,**36,**151

Formula: $C_{13} H_{11} Mo_1 O_3$

Compound Name: (η^5-(Cyclopenten-2-yl)cyclopentadienyl)-tricarbonyl-molybdenum

Space Group:	P-1	***Cell:***	***a***	7.517(1)	***b***	7.691(1)	***c***	11.303(2)
Space Group No.:	2	***(Å,°)***	α	87.72(0)	β	82.32(0)	γ	65.44(0)

R-Factor (%): 3.28 ***Temperature(K):*** 298 ***Density(g/cm^3):*** 1.755

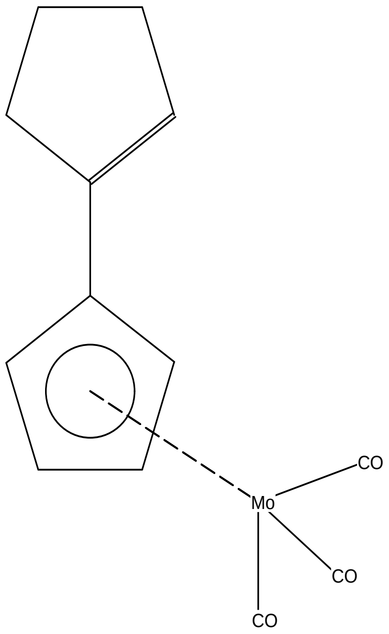

Parameters

Fragment 1

**DIST1 (D)**	1.384
**DIST2 (D)**	1.487
**DIST3 (D)**	1.515
**DIST4 (D)**	1.568
**DIST5 (D)**	1.506

LITBEV

Reference: He-Ping Wu, R.Aumann, R.Frohlich, B.Wibbeling (2000) *Eur.J.Org.Chem.* ,**2001,**1183

Formula: $C_{33} H_{32} O_9 W_1$

Compound Name: (1S,4R,7R)-3-(1,1,1,1,1-Pentacarbonyl-2-ethoxy-1-tungsta-2-ethynyl)-2-(cyclopent-1-enyl)-6-ethoxy-4-phenylacetoxy-tricyclo[5.3.0.0^{1,4}]deca-2,5-diene

Space Group:	P21/c	***Cell:***	***a***	17.924(3)	***b***	17.871(2)	***c***	9.944(2)
Space Group No.:	14	***(Å,°)***	α	90.00	β	98.17(1)	γ	90.00

R-Factor (%): 3.12 ***Temperature(K):*** 223 ***Density(g/cm^3):*** 1.594

Parameters

Fragment 1

**DIST1 (D)**	1.325
**DIST2 (D)**	1.502
**DIST3 (D)**	1.510
**DIST4 (D)**	1.485
**DIST5 (D)**	1.498

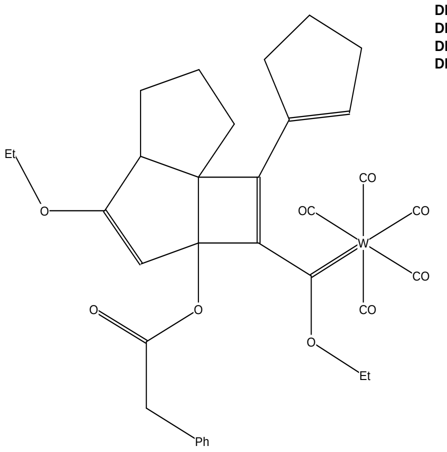

MALQIC

Reference: Huang Qiu, H.D.Srinivas, Peter Y.Zavalij, M.P.Doyle (2016) *J.Am.Chem.Soc.* ,**138,**1808

Formula: $C_{21} H_{17} Br_1 N_2 O_2$

Compound Name: 2-(cyclopent-1-en-1-yl)-1-phenylvinyl (4-bromophenyl)(diazo)acetate

Space Group:	C2/c	***Cell:***	***a***	37.080(2)	***b***	5.922(0)	***c***	16.784(1)
Space Group No.:	15	***(Å,°)***	α	90.00	β	98.63(0)	γ	90.00

R-Factor (%): 3.99 ***Temperature(K):*** 150 ***Density(g/cm^3):*** 1.492

Parameters

Fragment 1

**DIST1 (D)**	1.339
**DIST2 (D)**	1.475
**DIST3 (D)**	1.523
**DIST4 (D)**	1.475
**DIST5 (D)**	1.527

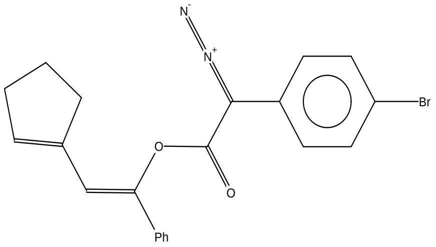

MUBLIE

Reference: A.Otaka, F.Katagiri, T.Kinoshita, Y.Odagaki, S.Oishi, H.Tamamura, N.Hamanaka, N.Fujii (2002) *J.Org.Chem.* ,**67,**6152

Formula: $C_{22} H_{35} N_1 O_4 S_1$

Compound Name: N-(1-(t-Butoxymethyl)-2-cyclopent-1-en-1-yl-3-hydroxypropyl)-2,4,6-trimethylbenzenesulfonamide

Space Group:	P212121	***Cell:***	***a***	13.361(6)	***b***	18.736(6)	***c***	9.330(30)
Space Group No.:	19	***(Å,°)***	α	90.00	β	90.00	γ	90.00

R-Factor (%): 9.18 ***Temperature(K):*** 296 ***Density(g/cm^3):*** 1.165

Parameters

Fragment 1

**DIST1 (D)**	1.268
**DIST2 (D)**	1.442
**DIST3 (D)**	1.617
**DIST4 (D)**	1.469
**DIST5 (D)**	1.493

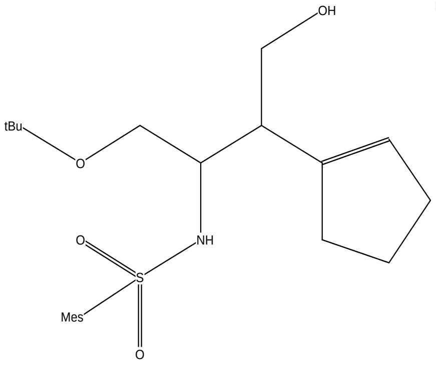

ONAKIY

Reference: Ki-Hyeok Kwon, D.W.Lee, C.S.Yi (2010) *Organometallics* , **29,**5748

Formula: $C_{37} H_{53} N_1 O_2 P_1 Ru_1{}^{1+}, B_1 F_4{}^{1-}, C_1 H_2 Cl_2$

Compound Name: Carbonyl-(3-(η^2-cyclopent-1-en-1-yl)-N,N-dimethyl-2-naphthamide)-hydrido-(tricyclohexylphosphine)-ruthenium tetrafluoroborate dichloromethane solvate

Space Group:	P-1	***Cell:***	***a*** 10.237(0)	***b*** 12.071(0)	***c*** 16.730(0)
Space Group No.:	2	***(Å,°)***	α 100.16(0)	β 102.32(0)	γ 103.84(0)

R-Factor (%): 2.14 ***Temperature(K):*** 100 ***Density(g/cm³):*** 1.479

Parameters

Fragment 1

**DIST1 (D)**	1.389
**DIST2 (D)**	1.523
**DIST3 (D)**	1.543
**DIST4 (D)**	1.540
**DIST5 (D)**	1.505

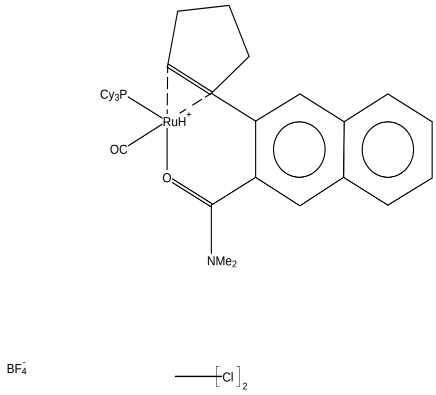

OXAMUX

Reference: K.O.Marichev, Huang Qiu, A.C.Offield, H.Arman, M.P.Doyle (2016) *J.Org.Chem.* ,**81,**9235

Formula: $C_{20} H_{18} N_2 O_1$

Compound Name: 3-(biphenyl-4-yl)-5-(cyclopent-1-en-1-yl)-1H-pyrazol-4-ol

Space Group:	Pna21	***Cell:***	***a*** 6.727(9)	***b*** 23.550(30)	***c*** 9.860(13)
Space Group No.:	33	***(Å,°)***	α 90.00	β 90.00	γ 90.00

R-Factor (%): 9.00 ***Temperature(K):*** 98 ***Density(g/cm³):*** 1.286

Parameters

Fragment 1

**DIST1 (D)**	1.355
**DIST2 (D)**	1.534
**DIST3 (D)**	1.586
**DIST4 (D)**	1.540
**DIST5 (D)**	1.534

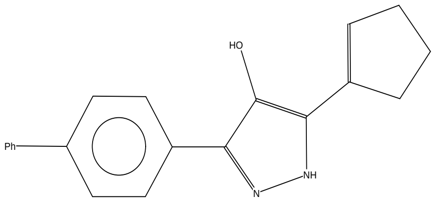

OXANAE

Reference: K.O.Marichev, Huang Qiu, A.C.Offield, H.Arman, M.P.Doyle (2016) *J.Org.Chem.* ,**81,**9235

Formula: $C_{21} H_{18} N_2 O_2$

Compound Name: 5-benzoyl-3-(cyclopent-1-en-1-yl)-5-phenyl-1,5-dihydro-4H-pyrazol-4-one

Space Group:	P21/c	***Cell:***	***a*** 17.920(7)	***b*** 6.022(2)	***c*** 15.745(6)
Space Group No.:	14	***(Å,°)***	α 90.00	β 99.30(0)	γ 90.00

R-Factor (%): 7.85 ***Temperature(K):*** 98 ***Density(g/cm³):*** 1.309

Parameters

Fragment 1

**DIST1 (D)**	1.327
**DIST2 (D)**	1.505
**DIST3 (D)**	1.523
**DIST4 (D)**	1.530
**DIST5 (D)**	1.493

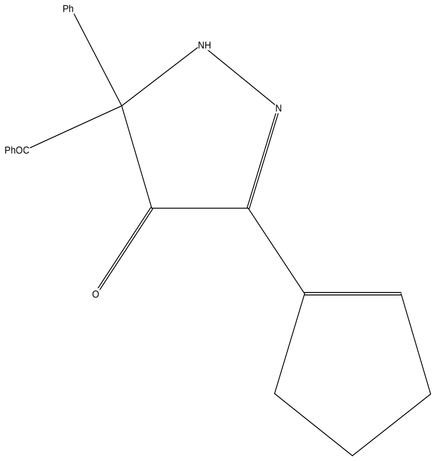

QEKGIZ

Reference: K.O.Marichev, E.C.Garcia, K.C.Bhowmick, D.J.Wherritt, H.Arman, M.P.Doyle (2017) *Chemical Science* ,**8,**7152

Formula: $C_{27} H_{22} N_2 O_2$

Compound Name: 3-([1,1'-biphenyl]-4-yl)-5-(cyclopent-1-en-1-yl)-1H-pyrazol-4-yl benzoate

Space Group:	P-1	***Cell:***	***a*** 5.743(1)	***b*** 12.655(3)	***c*** 15.118(4)
Space Group No.:	2	***(Å,°)***	α 69.39(0)	β 82.28(0)	γ 87.43(0)

R-Factor (%): 6.10 ***Temperature(K):*** 98 ***Density(g/cm³):*** 1.325

Parameters

Fragment 1

**DIST1 (D)**	1.340
**DIST2 (D)**	1.497
**DIST3 (D)**	1.529
**DIST4 (D)**	1.545
**DIST5 (D)**	1.496

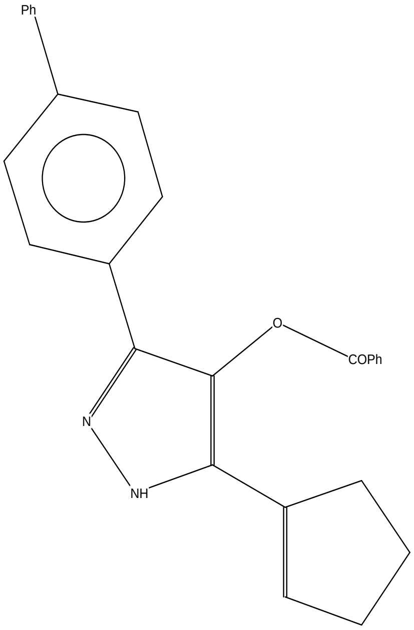

Search: search2 (Thu Mar 4 14:53:55 2021): Hits 25-28

RUCBEZ

Reference: Mintao Chen, Wenqing Zang, Yin Wei, Min Shi (2020) *Org.Biomol.Chem.* ,**18,**333

Formula: $C_{26} H_{29} N_3 O_6 S_1$

Compound Name: diethyl 1-{(cyclopent-1-en-1-yl)[1-(phenylsulfonyl)-1H-indol-3-yl]methyl}hydrazine-1,2-dicarboxylate

Space Group:	P21/n	***Cell:***	***a***	10.089(0)	***b***	23.696(0)	***c***	11.176(0)
Space Group No.:	14	***(Å,°)***	α	90.00	β	91.18(0)	γ	90.00

R-Factor (%): 5.88 ***Temperature(K):*** 296 ***Density(g/cm^3):*** 1.272

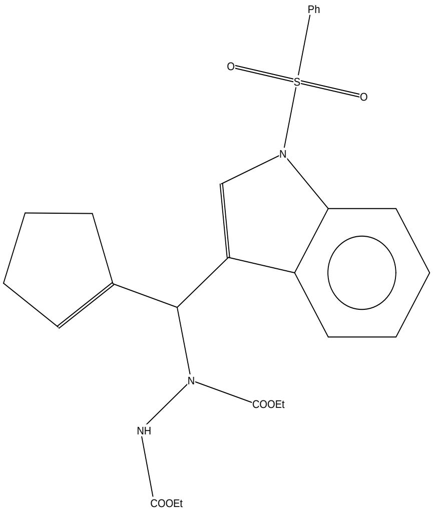

Parameters

Fragment 1

**DIST1 (D)**	1.330
**DIST2 (D)**	1.436
**DIST3 (D)**	1.529
**DIST4 (D)**	1.449
**DIST5 (D)**	1.500

SAFVAY

Reference: J.K.Lee, S.N.Chavre, Y.S.Cho, Y.Lee, J.H.Cha (2012) *Acta Crystallogr.,Sect.E:Struct.Rep.Online* ,**68,**o45

Formula: $C_{22} H_{21} Cl_1 O_2$

Compound Name: (2-(4-Chlorophenyl)-5-phenyltetrahydrofuran-3-yl)(cyclopent-1-en-1-yl) methanone

Space Group:	P21/n	***Cell:***	***a***	5.758(0)	***b***	11.355(1)	***c***	28.554(3)
Space Group No.:	14	***(Å,°)***	α	90.00	β	94.44(0)	γ	90.00

R-Factor (%): 5.07 ***Temperature(K):*** 296 ***Density(g/cm^3):*** 1.259

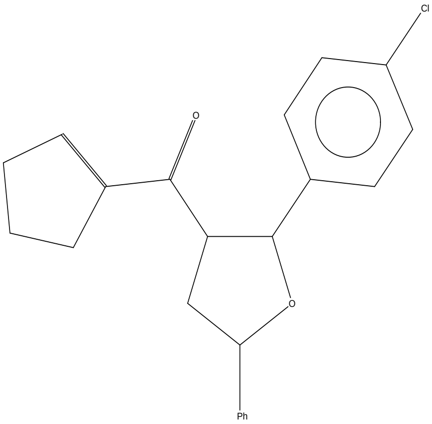

Parameters

Fragment 1

**DIST1 (D)**	1.317
**DIST2 (D)**	1.488
**DIST3 (D)**	1.505
**DIST4 (D)**	1.485
**DIST5 (D)**	1.497

SUSNUP

Reference: V.Nair, J.S.Nair, S.Kumar, N.P.Rath, P.G.Williard, G.K.Eigendorf (1998) *Tetrahedron Lett.* ,**39,**4603

Formula: $C_{39} H_{32} O_1$

Compound Name: 4-Cyclopentenyl-1,7,8,9-tetraphenyltricyclo(5.2.1.0^{2,6})deca-3,8-dien-10-one

Space Group:	P-1	***Cell:***	***a***	9.462(1)	***b***	12.626(1)	***c***	13.569(2)
Space Group No.:	2	***(Å,°)***	α	63.97(1)	β	84.12(1)	γ	87.07(1)

R-Factor (%): 5.61 ***Temperature(K):*** 295 ***Density(g/cm^3):*** 1.184

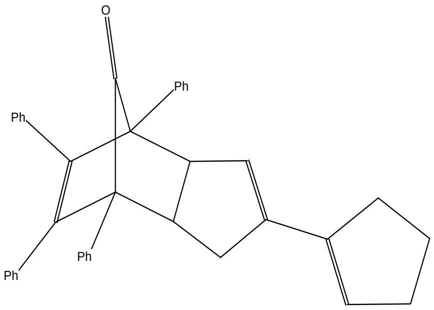

Parameters

Fragment 1

**DIST1 (D)**	1.388
**DIST2 (D)**	1.433
**DIST3 (D)**	1.492
**DIST4 (D)**	1.497
**DIST5 (D)**	1.504

SUSPAX

Reference: V.Nair, J.S.Nair, S.Kumar, N.P.Rath, P.G.Williard, G.K.Eigendorf (1998) *Tetrahedron Lett.* ,**39,**4603

Formula: $C_{26} H_{24} N_4$

Compound Name: 1-Cyclopentyl-4,4,5,5-tetracyano-pentacyclo(12.2.1.0^{2,13}.0^{3,11}.0^{6,10}) heptadeca-10,15-diene

Space Group:	P21/n	***Cell:***	***a***	7.850(2)	***b***	18.988(4)	***c***	14.065(3)
Space Group No.:	14	***(Å,°)***	α	90.00	β	90.43	γ	90.00

R-Factor (%): 14.04 ***Temperature(K):*** 295 ***Density(g/cm^3):*** 1.244

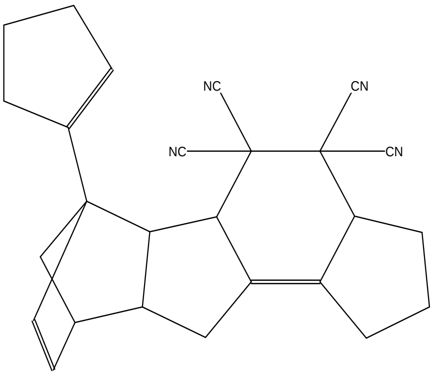

Parameters

Fragment 1

**DIST1 (D)**	1.318
**DIST2 (D)**	1.492
**DIST3 (D)**	1.527
**DIST4 (D)**	1.480
**DIST5 (D)**	1.508

Search: search2 (Thu Mar 4 14:53:55 2021): Hits 29-32

TEYGAF

Reference: K.Kakiuchi, H.Okada, N.Kanehisa, Y.Kai, H.Kurosawa (1996) *J.Org.Chem.* ,**61,**2972

Formula: $C_{21}\ H_{31}\ Cl_{1}$

Compound Name: 7-Chloro-2-cyclopentenyltetracyclo(5.3.3.3^{2,6}.0^{1,6})hexadecane

Space Group: P21/n ***Cell:*** ***a*** 12.371(2) ***b*** 11.642(2) ***c*** 12.708(2)
Space Group No.: 14 ***(Å,°)*** α 90.00 β 112.49(0) γ 90.00

R-Factor (%): 5.00 ***Temperature(K):*** 295 ***Density(g/cm^3):*** 1.253

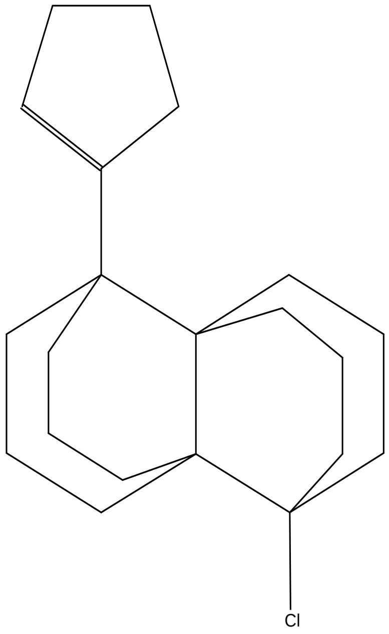

Parameters

Fragment 1

**DIST1 (D)**	1.382
**DIST2 (D)**	1.459
**DIST3 (D)**	1.505
**DIST4 (D)**	1.488
**DIST5 (D)**	1.489

TOBSUA

Reference: Hua-Rong Zhao, Ling Wang (2014) *Jiegou Huaxue(Chin.)(Chin.J.Struct.Chem.)* ,**33,**284

Formula: $C_{15}\ H_{18}\ N_{2}\ O_{1}\ S_{1}$

Compound Name: N-((2-Phenylethyl)carbamothioyl)cyclopent-1-ene-1-carboxamide

Space Group: P-1 ***Cell:*** ***a*** 6.950(0) ***b*** 9.462(0) ***c*** 11.326(1)
Space Group No.: 2 ***(Å,°)*** α 71.52(0) β 81.83(0) γ 89.24(0)

R-Factor (%): 4.13 ***Temperature(K):*** 293 ***Density(g/cm^3):*** 1.304

Parameters

Fragment 1

**DIST1 (D)**	1.325
**DIST2 (D)**	1.498
**DIST3 (D)**	1.529
**DIST4 (D)**	1.490
**DIST5 (D)**	1.495

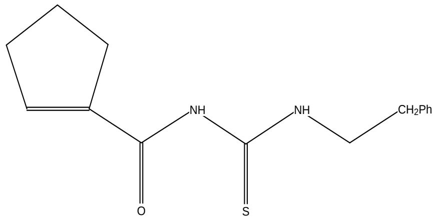

TUMDOU

Reference: J.Barluenga, F.Aznar, B.Weyershausen, S.Garcia-Granda, E.Martin (1996) *Chem.Commun.* ,2455

Formula: $C_{22}\ H_{22}\ N_{2}\ O_{6}\ W_{1}$

Compound Name: ((1-Cyclopentenyl)(N-morpholino)methylidene)-(1-methyl-3-oxa-indoline-2-ylidene)-tetracarbonyl-tungsten(0)

Space Group: P21/c ***Cell:*** ***a*** 16.163(9) ***b*** 8.346(2) ***c*** 16.643(8)
Space Group No.: 14 ***(Å,°)*** α 90.00 β 99.87(5) γ 90.00

R-Factor (%): 3.90 ***Temperature(K):*** 295 ***Density(g/cm^3):*** 1.785

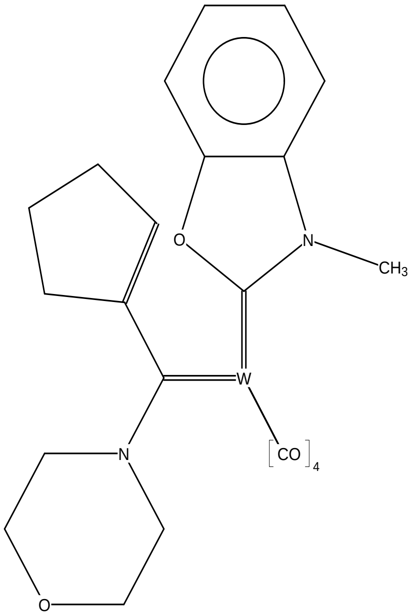

Parameters

Fragment 1

**DIST1 (D)**	1.365
**DIST2 (D)**	1.445
**DIST3 (D)**	1.494
**DIST4 (D)**	1.486
**DIST5 (D)**	1.507

UBIHOC

Reference: F.Lieb, J.Benet-Buchholz, T.Facke, R.Fischer, A.Graff, I.M.Lefebvre, J.Stetter (2001) *Tetrahedron* ,**57,**4133

Formula: $C_{19}\ H_{22}\ O_{3}$

Compound Name: 5-(1-Cyclopenten-1-yl)-4-hydroxy-3-mesityl-5-methyl-2(5H)-furanone

Space Group: P21/c ***Cell:*** ***a*** 11.715(0) ***b*** 12.327(0) ***c*** 11.384(0)
Space Group No.: 14 ***(Å,°)*** α 90.00 β 91.05(0) γ 90.00

R-Factor (%): 4.69 ***Temperature(K):*** 153 ***Density(g/cm^3):*** 1.206

Parameters

Fragment 1

**DIST1 (D)**	1.324
**DIST2 (D)**	1.515
**DIST3 (D)**	1.528
**DIST4 (D)**	1.542
**DIST5 (D)**	1.507

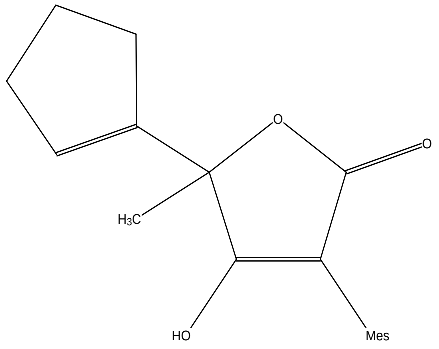

Search: search2 (Thu Mar 4 14:53:55 2021): Hits 33-36

UQAKIH

Reference: Zhi-Hong Ma, Xiao-Huan Liu, Li-Zhi Lin, Zhan-Gang Han, Xue-Zhong Zheng, Jin Lin (2011) *Wuji Huaxue Xuebao(Chin.)(Chin.J.Inorg.Chem.)* ,**27,**913

Formula: $C_{24} H_{22} O_4 Ru_2$

Compound Name: bis(μ_2-Carbonyl)-bis(η^5-1-cyclopentenylcyclopentadienyl)-dicarbonyl-di-ruthenium(i)

Space Group:	P-1	***Cell:***	***a***	6.416(1)	***b***	7.613(2)	***c***	11.924(3)
Space Group No.:	2	***(Å,°)***	α	89.97(0)	β	87.14(0)	γ	69.67(0)

R-Factor (%): 2.43 ***Temperature(K):*** 298 ***Density(g/cm^3):*** 1.756

Parameters

Fragment 1

**DIST1 (D)**	1.389
**DIST2 (D)**	1.432
**DIST3 (D)**	1.513
**DIST4 (D)**	1.481
**DIST5 (D)**	1.505

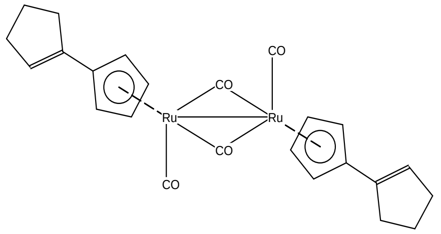

WIDBAP

Reference: Teng Liu, Xue Sun, Lei Wu (2018) *Adv.Synth.Catal.* ,**360,** 2005

Formula: $C_{20} H_{19} O_1 P_1$

Compound Name: 3-(cyclopent-1-en-1-yl)-2-methyl-1-phenyl-1H-1λ^5-phosphindol-1-one

Space Group:	P212121	***Cell:***	***a***	9.627(0)	***b***	12.989(0)	***c***	13.108(0)
Space Group No.:	19	***(Å,°)***	α	90.00	β	90.00	γ	90.00

R-Factor (%): 4.13 ***Temperature(K):*** 296 ***Density(g/cm^3):*** 1.242

Parameters

Fragment 1

**DIST1 (D)**	1.336
**DIST2 (D)**	1.464
**DIST3 (D)**	1.501
**DIST4 (D)**	1.510
**DIST5 (D)**	1.484

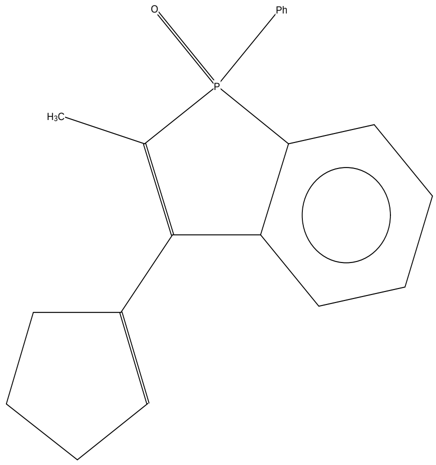

WIMRUH

Reference: R.Kotikalapudi, K.C.Kumara Swamy (2013) *Tetrahedron* , **69,**8002

Formula: $C_{21} H_{25} O_4 P_1$

Compound Name: 2-(3-Benzyl-5-(cyclopent-1-en-1-yl)-2-furyl)-5,5-dimethyl-1,3,2-dioxaphosphinane 2-oxide

Space Group:	P21	***Cell:***	***a***	6.533(4)	***b***	9.469(5)	***c***	16.085(10)
Space Group No.:	4	***(Å,°)***	α	90.00	β	96.07(0)	γ	90.00

R-Factor (%): 3.61 ***Temperature(K):*** 298 ***Density(g/cm^3):*** 1.250

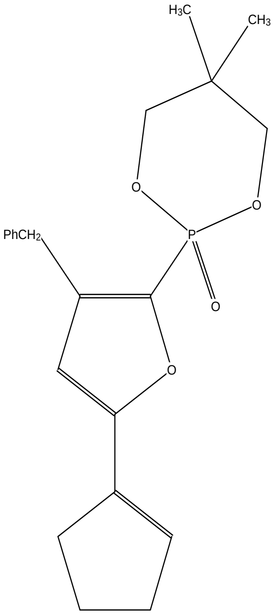

Parameters

Fragment 1

**DIST1 (D)**	1.325
**DIST2 (D)**	1.503
**DIST3 (D)**	1.521
**DIST4 (D)**	1.498
**DIST5 (D)**	1.501

XEDRAZ

Reference: He-Ping Wu, R.Aumann, S.Venne-Dunker, P.Saarenketo (2000) *Eur.J.Org.Chem.* ,**2001,**3463

Formula: $C_{31} H_{30} O_7 S_1 W_1$

Compound Name: (1S,4R,7R)-2-(Cyclopent-1-enyl)-6-ethoxy-3-(1,1,1,1,1-pentacarbonyl-2-ethoxy-1-tungsta-2-ethenyl)-4-phenylthiotricyclo(5.3.0.0^{1,4})deca-2,5-diene

Space Group:	P21/c	***Cell:***	***a***	15.558(1)	***b***	18.648(1)	***c***	10.051(1)
Space Group No.:	14	***(Å,°)***	α	90.00	β	94.49(1)	γ	90.00

R-Factor (%): 3.26 ***Temperature(K):*** 198 ***Density(g/cm^3):*** 1.669

Parameters

Fragment 1

**DIST1 (D)**	1.332
**DIST2 (D)**	1.505
**DIST3 (D)**	1.539
**DIST4 (D)**	1.506
**DIST5 (D)**	1.497

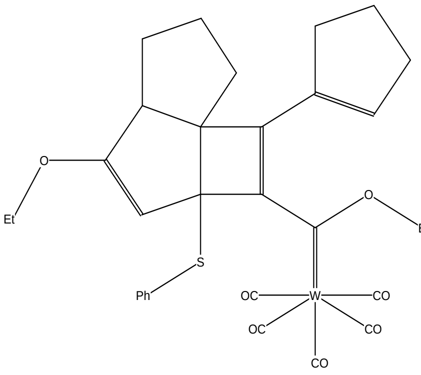

Search: search2 (Thu Mar 4 14:53:55 2021): Hits 37-40

XIRNOB

Reference: P.-J.Sinnema, P.J.Shapiro, B.Hohn, T.E.Bitterwolf, B.Twamley (2001) *Organometallics* ,**20,**2883

Formula: $C_{28}\ H_{38}\ Ca_1\ O_2$

Compound Name: bis(η^5-1-(Cyclopentenyl)cyclopentadienyl)-bis(tetrahydrofuran)-calcium

Space Group: C2/c ***Cell:*** ***a*** 38.335(0) ***b*** 8.264(0) ***c*** 16.160(0)
Space Group No.: 15 ***(Å,°)*** α 90.00 β 98.48(0) γ 90.00

R-Factor (%): 7.03 ***Temperature(K):*** 203 ***Density(g/cm³):*** 1.172

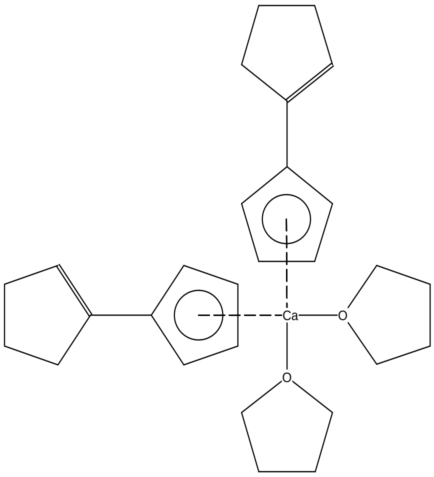

Parameters

Fragment 1

**DIST1 (D)**	1.399
**DIST2 (D)**	1.422
**DIST3 (D)**	1.513
**DIST4 (D)**	1.487
**DIST5 (D)**	1.501

Fragment 2

**DIST1 (D)**	1.417
**DIST2 (D)**	1.430
**DIST3 (D)**	1.511
**DIST4 (D)**	1.506
**DIST5 (D)**	1.508

YETJOW

Reference: J.Barluenga, F.Aznar, A.Martin, S.Garcia-Granda, E.Perez-Carreno (1994) *J.Am.Chem.Soc.* ,**116,**11191

Formula: $C_{14}\ H_{15}\ Cr_1\ N_1\ O_5$

Compound Name: Tetracarbonyl-((η^2-1-cyclopentenyl)-(N-morpholino)-methylene)-chromium(0)

Space Group: P21/n ***Cell:*** ***a*** 6.667(1) ***b*** 12.412(3) ***c*** 17.821(3)
Space Group No.: 14 ***(Å,°)*** α 90.00 β 94.18(2) γ 90.00

R-Factor (%): 2.90 ***Temperature(K):*** 200 ***Density(g/cm³):*** 1.487

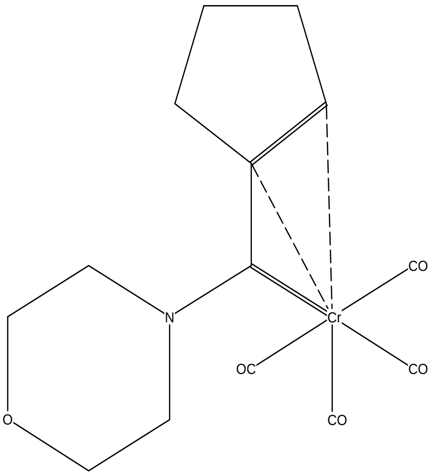

Parameters

Fragment 1

**DIST1 (D)**	1.367
**DIST2 (D)**	1.518
**DIST3 (D)**	1.533
**DIST4 (D)**	1.529
**DIST5 (D)**	1.507

YOXLUS

Reference: H.-J.Gais, H.Muller, J.Bund, M.Scommoda, J.Brandt, G.Raabe (1995) *J.Am.Chem.Soc.* ,**117,**2453

Formula: $C_{19}\ H_{21}\ N_1\ O_3\ S_2$

Compound Name: (+)-(S)-S-(1-Cyclopenten-1-ylmethyl)-N-(p-tolylsulfonyl)-S-phenylsulfoximine

Space Group: P21 ***Cell:*** ***a*** 8.807(1) ***b*** 12.021(1) ***c*** 8.939(1)
Space Group No.: 4 ***(Å,°)*** α 90.00 β 98.67(0) γ 90.00

R-Factor (%): 3.70 ***Temperature(K):*** 295 ***Density(g/cm³):*** 1.333

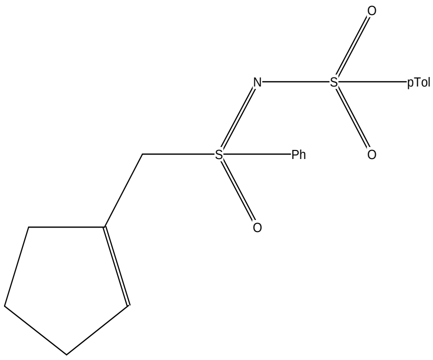

Parameters

Fragment 1

**DIST1 (D)**	1.344
**DIST2 (D)**	1.465
**DIST3 (D)**	1.520
**DIST4 (D)**	1.490
**DIST5 (D)**	1.514

ZICNAD

Reference: A.Saikia (2018) *CSD Communication(Private Communication) ,*

Formula: $C_{16}\ H_{17}\ N_1\ O_3$

Compound Name: 5-(cyclopent-1-en-1-yl)-6-(2-nitrophenyl)-3,6-dihydro-2H-pyran

Space Group: P-1 ***Cell:*** ***a*** 7.353(1) ***b*** 12.219(2) ***c*** 15.266(3)
Space Group No.: 2 ***(Å,°)*** α 97.16(1) β 90.23(1) γ 90.15(1)

R-Factor (%): 5.20 ***Temperature(K):*** 296 ***Density(g/cm³):*** 1.324

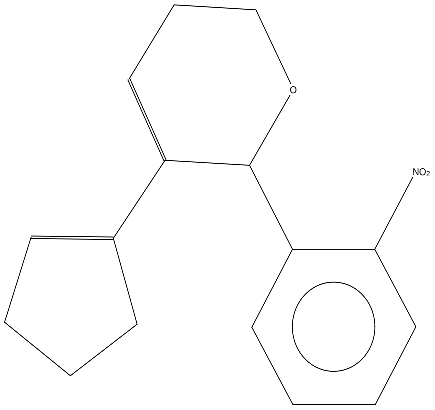

Parameters

Fragment 1

**DIST1 (D)**	1.324
**DIST2 (D)**	1.498
**DIST3 (D)**	1.530
**DIST4 (D)**	1.522
**DIST5 (D)**	1.482

Fragment 2

**DIST1 (D)**	1.326
**DIST2 (D)**	1.499
**DIST3 (D)**	1.530
**DIST4 (D)**	1.522
**DIST5 (D)**	1.484
