# Supplementary material for: The crystal structures, Hirshfeld surface analyses and energy frameworks of 8-{1-[3-(cyclo­pent-1-en-1-yl)benz­yl]piperidin-4-yl}-2-meth­oxy­quinoline and 8-{4-[3-(cyclo­pent-1-en-1-yl)benz­yl]piperazin-1-yl}-2-meth­oxy­quinoline
Source: Acta Crystallogr E Crystallogr Commun. 2021 Mar 9;77(Pt 4):372–7. doi: 10.1107/S2056989021002474 (PMC8025868; doi:10.1107/S2056989021002474)
Supplement: Supplementary file 7 [file e-77-00372-sup7.pdf]

Compound III [Ullah, N. & Altaf, M. (2014). Crystallogr. Rep. 59, 1057-1062]

CCDC 987772, CSD refcode AKUXIQ

Supplementary Table S1

Hydrogen-bond geometry (Å, °)

CgA is the centroid of ring N1/C1-C4/C9 ; CgD is the centroid of phenyl ring C15-C20.

D—H···*A*	*D*—H	H···*A*	*D*···*A*	*D*—H···*A*
C24—H24···O1^{i}	0.95	2.51	3.344 (3)	146
C27—H27*B*···CgA^{ii}	0.98	2.88	3.635 (2)	134
C6—H6···CgD^{iii}	0.95	2.79	3.619 (2)	146

Symmetry codes: (i) *x*+1, *y*, *z*; (ii) *x*, *y*+1, *z*; (iii) −*x*+2, −*y*+1, −*z*+2.

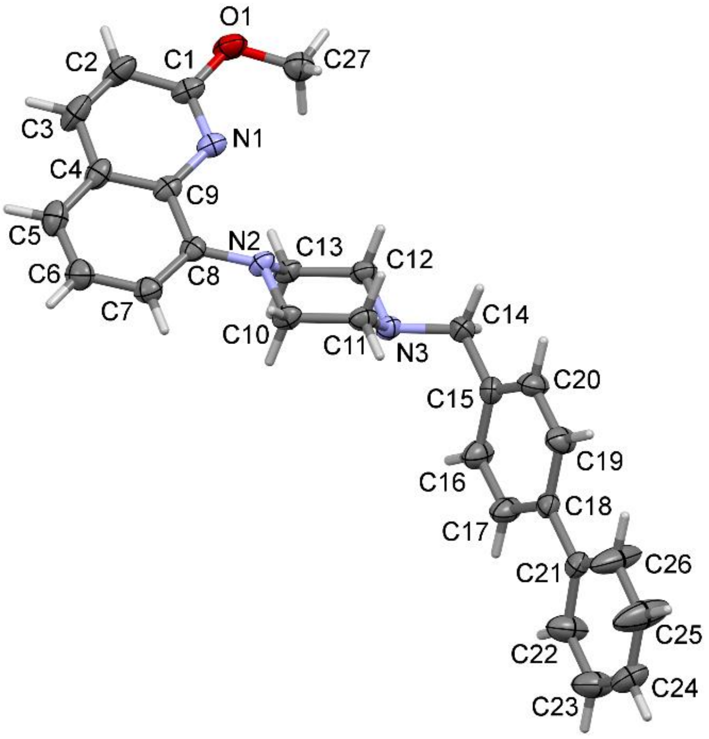

Fig S1. A view of the molecular structure of compound **III**, with atom labelling. Displacement ellipsoids are drawn at the 50% probabilty level.

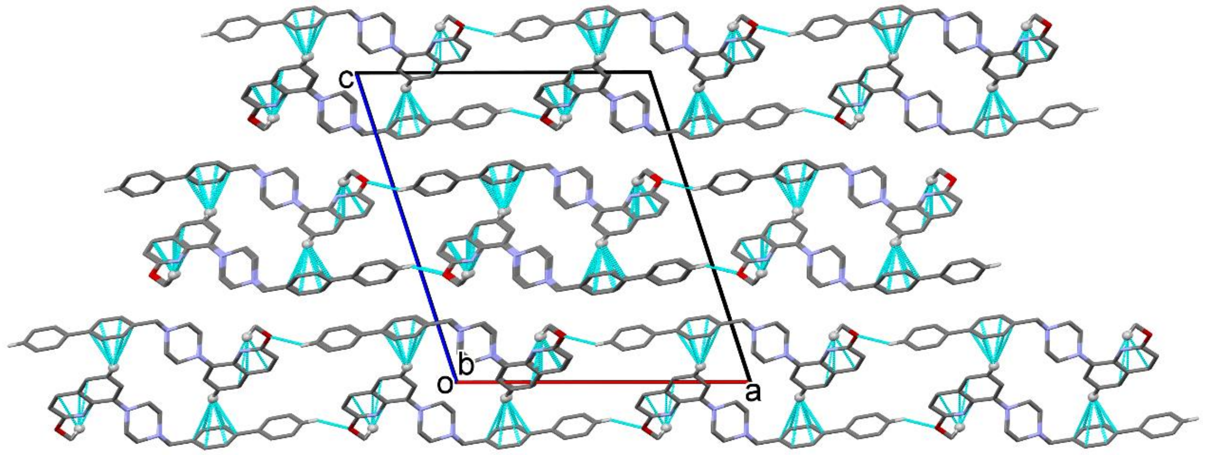

Fig. S2. A view along the *b* axis of the crystal packing of compound **III**. Only the H atoms involved in the C-H…O hydrogen bonds and the C-H…π interactions (dashed lines – see Table S1 above) have been included
