# Supplementary material for: The crystal structures, Hirshfeld surface analyses and energy frameworks of 8-{1-[3-(cyclo­pent-1-en-1-yl)benz­yl]piperidin-4-yl}-2-meth­oxy­quinoline and 8-{4-[3-(cyclo­pent-1-en-1-yl)benz­yl]piperazin-1-yl}-2-meth­oxy­quinoline
Source: Acta Crystallogr E Crystallogr Commun. 2021 Mar 9;77(Pt 4):372–7. doi: 10.1107/S2056989021002474 (PMC8025868; doi:10.1107/S2056989021002474)
Supplement: Supplementary file 8 [file e-77-00372-sup8.pdf]

**Supplementary file S2**

**The colour-coded interaction mappings within a radius of 6 Å of a central reference molecule for compounds (a) I, (b) II and (c) III, are given below.**
**Full details of the various contributions to the total energy ($E_{tot}$) are also given.**

## (a) I

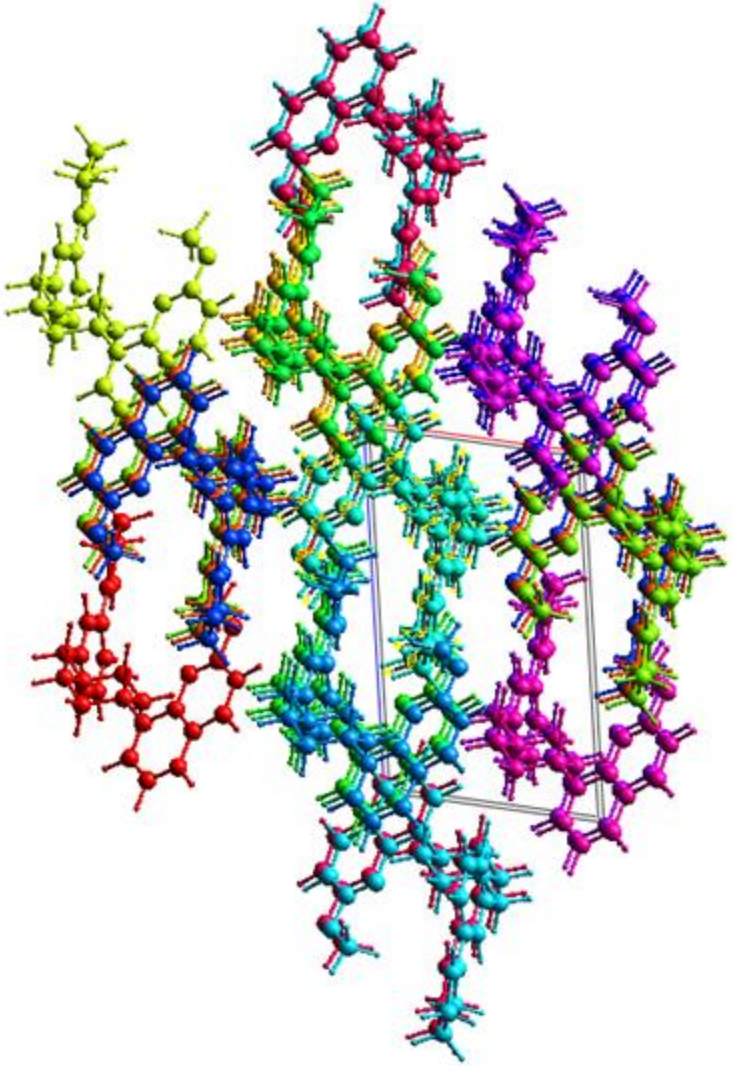

| | N | Symop | R | Electron Density | E_ele | E_pol | E_dis | E_rep | E_tot |
|---|---|---|---|---|---|---|---|---|---|
| | 1 | -x, -y, -z | 11.24 | HF/3-21G | -1.1 | -0.2 | -4.2 | 0.5 | -4.6 |
| | 2 | x, y, z | 7.71 | HF/3-21G | -3.2 | -0.7 | -30.6 | 12.9 | -20.8 |
| | 1 | -x, -y, -z | 15.76 | HF/3-21G | -6.6 | -1.2 | -16.3 | 7.4 | -16.2 |
| | 1 | -x, -y, -z | 7.74 | HF/3-21G | -2.7 | -3.1 | -53.2 | 20.4 | -36.1 |
| | 1 | -x, -y, -z | 19.71 | HF/3-21G | 0.6 | -0.0 | -0.5 | 0.0 | 0.1 |
| | 2 | x, y, z | 14.50 | HF/3-21G | -7.4 | -2.2 | -19.4 | 9.4 | -18.9 |
| | 1 | -x, -y, -z | 6.88 | HF/3-21G | -5.4 | -1.8 | -45.5 | 18.6 | -32.7 |
| | 1 | -x, -y, -z | 11.23 | HF/3-21G | -0.2 | -0.0 | -1.5 | 0.0 | -1.6 |
| | 1 | -x, -y, -z | 10.79 | HF/3-21G | -8.3 | -2.7 | -56.5 | 22.0 | -43.3 |
| | 2 | x, y, z | 11.28 | HF/3-21G | -4.9 | -1.8 | -38.1 | 15.5 | -27.9 |
| | 2 | x, y, z | 17.27 | HF/3-21G | 0.0 | -0.2 | -5.6 | 2.7 | -2.9 |
| | 1 | -x, -y, -z | 20.98 | HF/3-21G | 0.1 | -0.0 | -0.6 | 0.0 | -0.5 |
| | 2 | x, y, z | 12.78 | HF/3-21G | 0.1 | -0.0 | -0.9 | 0.0 | -0.7 |
| | 1 | -x, -y, -z | 7.87 | HF/3-21G | -17.1 | -4.1 | -64.7 | 34.9 | -50.1 |
| | 1 | -x, -y, -z | 15.07 | HF/3-21G | 0.1 | -0.0 | -1.3 | 0.0 | -1.1 |
| | 1 | -x, -y, -z | 9.33 | HF/3-21G | 0.3 | -0.0 | -2.9 | 0.0 | -2.3 |
| | 1 | -x, -y, -z | 12.31 | HF/3-21G | -0.8 | -0.1 | -5.3 | 0.3 | -5.3 |
| | 1 | -x, -y, -z | 13.41 | HF/3-21G | -5.9 | -1.9 | -39.4 | 12.1 | -32.9 |
| | 2 | x, y, z | 12.95 | HF/3-21G | -0.2 | -0.0 | -0.8 | 0.0 | -0.9 |

**(b) II**

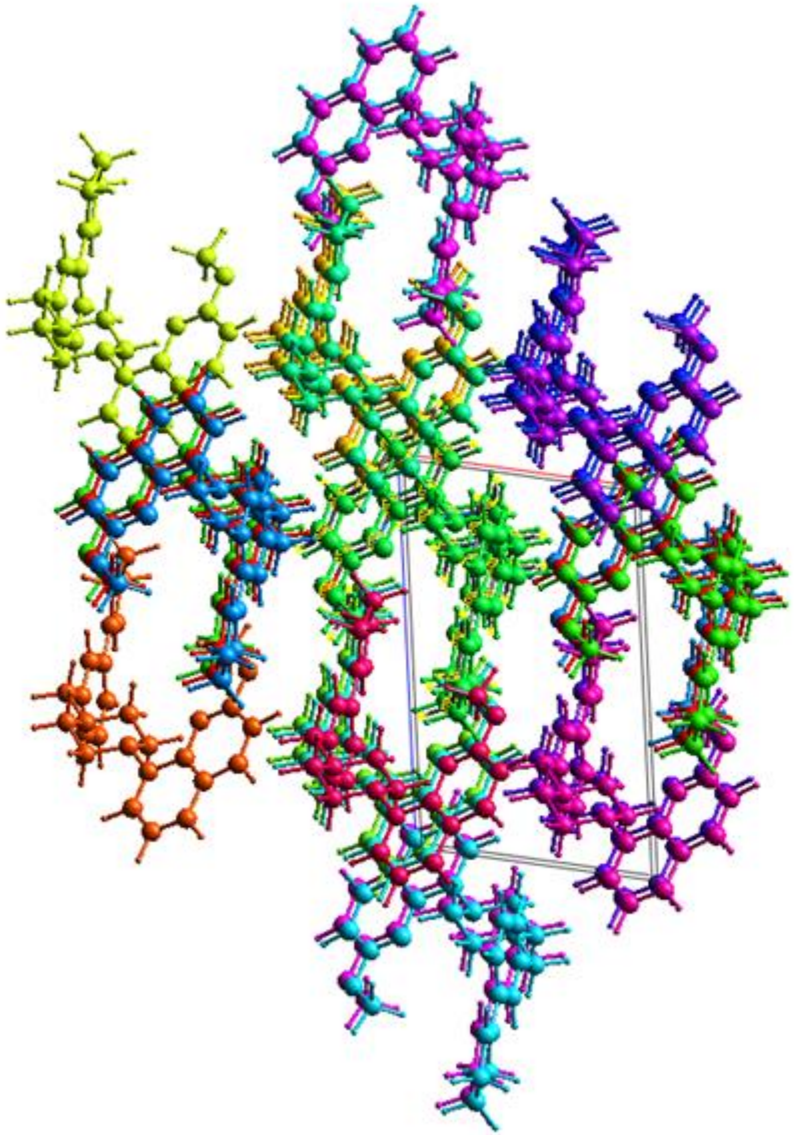

| | N | Symop | R | Electron Density | E_ele | E_pol | E_dis | E_rep | E_tot |
|---|---|---|---|---|---|---|---|---|---|
| | 2 | x, y, z | 7.91 | HF/3-21G | -4.2 | -0.9 | -30.8 | 14.2 | -21.1 |
| | 1 | -x, -y, -z | 11.36 | HF/3-21G | -1.1 | -0.1 | -3.6 | 0.3 | -4.2 |
| | 1 | -x, -y, -z | 15.47 | HF/3-21G | -7.5 | -1.4 | -18.1 | 10.0 | -16.7 |
| | 1 | -x, -y, -z | 7.66 | HF/3-21G | -5.6 | -3.1 | -57.6 | 23.3 | -40.8 |
| | 1 | -x, -y, -z | 19.58 | HF/3-21G | 0.6 | -0.0 | -0.5 | 0.0 | 0.1 |
| | 1 | -x, -y, -z | 6.81 | HF/3-21G | -7.1 | -2.4 | -47.7 | 20.5 | -35.1 |
| | 2 | x, y, z | 14.32 | HF/3-21G | -6.6 | -2.0 | -18.4 | 8.5 | -17.7 |
| | 2 | x, y, z | 10.91 | HF/3-21G | -9.7 | -3.3 | -39.2 | 16.7 | -33.8 |
| | 1 | -x, -y, -z | 10.76 | HF/3-21G | -0.3 | -0.0 | -1.7 | 0.0 | -1.8 |
| | 1 | -x, -y, -z | 10.81 | HF/3-21G | -7.3 | -2.4 | -54.1 | 19.0 | -42.3 |
| | 2 | x, y, z | 17.28 | HF/3-21G | 0.1 | -0.2 | -5.7 | 2.8 | -2.9 |
| | 2 | x, y, z | 12.58 | HF/3-21G | 0.1 | -0.0 | -0.9 | 0.0 | -0.7 |
| | 1 | -x, -y, -z | 7.96 | HF/3-21G | -14.7 | -2.9 | -58.0 | 32.1 | -43.1 |
| | 1 | -x, -y, -z | 14.85 | HF/3-21G | -0.3 | -0.0 | -1.3 | 0.0 | -1.5 |
| | 1 | -x, -y, -z | 9.43 | HF/3-21G | 0.3 | -0.1 | -3.0 | 0.0 | -2.5 |
| | 1 | -x, -y, -z | 12.00 | HF/3-21G | -0.8 | -0.1 | -5.5 | 0.4 | -5.5 |
| | 2 | x, y, z | 12.89 | HF/3-21G | -0.2 | -0.0 | -0.8 | 0.0 | -1.0 |
| | 1 | -x, -y, -z | 13.53 | HF/3-21G | -4.7 | -1.6 | -33.5 | 7.4 | -29.9 |
| | 1 | -x, -y, -z | 20.62 | HF/3-21G | 0.1 | -0.0 | -0.7 | 0.0 | -0.6 |

**(c) III**

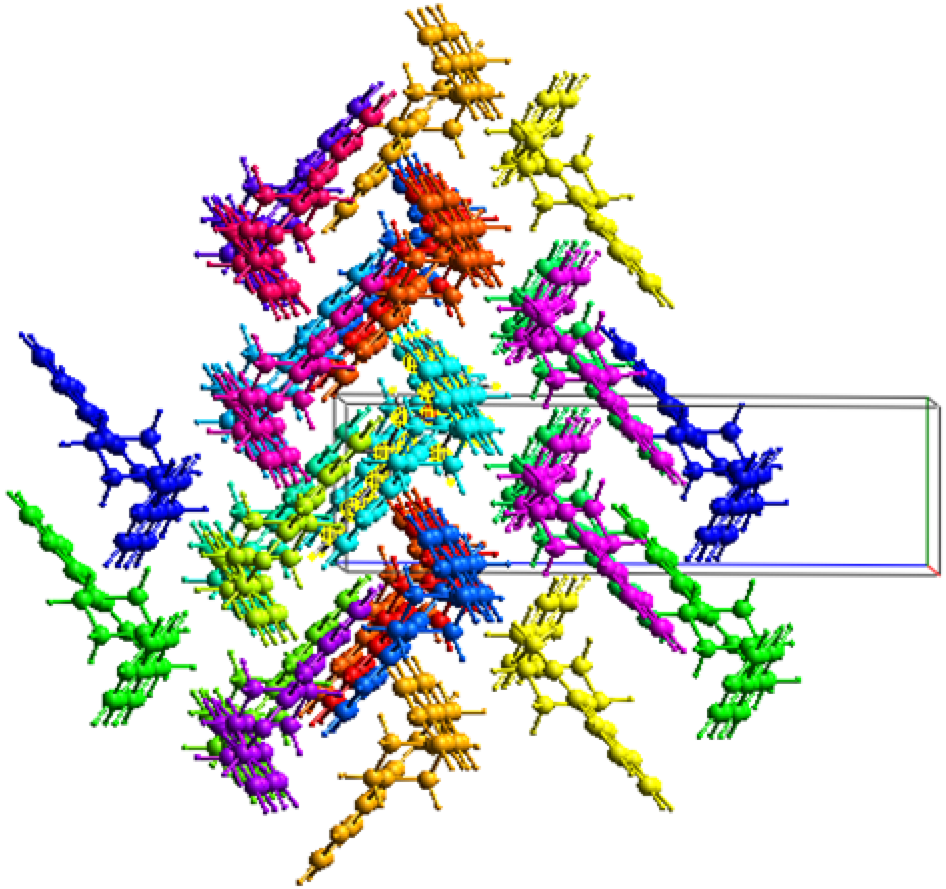

| | N | Symop | R | Electron Density | E_ele | E_pol | E_dis | E_rep | E_tot |
|---|---|---|---|---|---|---|---|---|---|
| | 2 | x, y, z | 5.69 | HF/3-21G | -8.0 | -4.0 | -68.9 | 26.3 | -51.5 |
| | 2 | x, y, z | 19.84 | HF/3-21G | 0.7 | -0.5 | -8.2 | 3.2 | -4.4 |
| | 2 | x, y, z | 22.16 | HF/3-21G | 0.2 | -0.0 | -0.3 | 0.0 | -0.0 |
| | 2 | -x+1/2, y+1/2, -z+1/2 | 11.54 | HF/3-21G | -0.6 | -0.0 | -1.7 | 0.0 | -2.2 |
| | 1 | -x, -y, -z | 6.19 | HF/3-21G | -16.0 | -5.6 | -72.4 | 36.8 | -55.3 |
| | 1 | -x, -y, -z | 21.36 | HF/3-21G | 0.5 | -0.0 | -0.4 | 0.0 | 0.2 |
| | 2 | x+1/2, -y+1/2, z+1/2 | 17.54 | HF/3-21G | -0.0 | -0.0 | -0.6 | 0.0 | -0.5 |
| | 2 | -x+1/2, y+1/2, -z+1/2 | 8.27 | HF/3-21G | -4.6 | -2.1 | -44.4 | 20.4 | -29.5 |
| | 1 | -x, -y, -z | 19.49 | HF/3-21G | -0.3 | -0.1 | -1.8 | 0.0 | -1.9 |
| | 2 | x, y, z | 19.01 | HF/3-21G | -7.2 | -1.8 | -9.8 | 6.1 | -12.4 |
| | 1 | -x, -y, -z | 5.16 | HF/3-21G | -3.9 | -4.5 | -69.9 | 29.9 | -45.7 |
| | 2 | x, y, z | 19.84 | HF/3-21G | 0.1 | -0.0 | -0.4 | 0.0 | -0.3 |
| | 2 | x+1/2, -y+1/2, z+1/2 | 11.87 | HF/3-21G | 0.2 | -0.0 | -1.0 | 0.0 | -0.7 |
| | 1 | -x, -y, -z | 8.93 | HF/3-21G | -1.5 | -0.1 | -3.1 | 0.0 | -4.3 |
| | 1 | -x, -y, -z | 10.71 | HF/3-21G | -0.5 | -0.0 | -2.0 | 0.0 | -2.4 |
| | 2 | -x+1/2, y+1/2, -z+1/2 | 14.53 | HF/3-21G | -6.1 | -2.5 | -31.3 | 12.3 | -26.1 |
| | 1 | -x, -y, -z | 20.20 | HF/3-21G | -0.7 | -0.1 | -1.4 | 0.0 | -2.0 |
| | 1 | -x, -y, -z | 21.47 | HF/3-21G | 0.3 | -0.0 | -0.9 | 0.0 | -0.6 |
